# Impact of Bacillus Calmette-Guérin (BCG) vaccination on postoperative mortality in patients with perioperative SARS-CoV-2 infection

**DOI:** 10.1093/bjsopen/zrab131

**Published:** 2022-01-08

**Authors:** COVID Surg Collaborative

## Abstract

There is little evidence around the potentially protective role of previous Bacillus Calmette-Guerin (BCG) vaccination on postoperative mortality in patients with perioperative SARS-CoV-2 vaccination. Prior BCG vaccination did not protect SARS-CoV-2 infected patients against postoperative pulmonary complications and 30-day mortality.

*Dear Editor*

During the COVID-19 pandemic, multiple theories were proposed based on the observation that countries with an ongoing national Bacillus Calmette-Guérin (BCG) immunization programme had lower SARS-CoV-2 case rates and COVID-19 mortality than countries that had stopped BCG vaccine administration. There was, however, no evidence supporting or disputing this theory^1^^,^^2^. Owing to the uncertainty on the role of BCG vaccine on the outcomes of patients with perioperative SARS-CoV-2 infection, this international study aimed to determine whether previous BCG vaccination was associated with reduced postoperative pulmonary complications (PPC) and 30-day mortality in patients undergoing surgery who developed SARS-CoV-2 infection.

This COVIDSurg study^3–5^ was an international prospective cohort study that included patients who had surgery between 1 February and 31 July 2020 and were diagnosed with SARS-CoV-2 within 7 days before or 30 days after surgery. The primary outcome was the rate of 30-day PPC and the secondary outcome was 30-day postoperative mortality rate. These were compared in patients with and without BCG vaccination. A logistic regression model was used to find the odds of association. The full methodology is available in the *Appendix S2*.

This study included 2391 patients from 428 hospitals across 64 countries from February 2020 to July 2020. Overall, 1165 (48.9 per cent) patients were male and 524 (21.9 per cent) were aged 70 years or older (*Table S1*). The BCG vaccine had been administered to 1649 (69.0 per cent) patients, of whom 1518 (63.5 per cent) had received it at least 15 years before surgery. Patients who had received a BCG vaccination were younger than patients who had not been vaccinated (70 years of age or older: 16 *versus* 35 per cent (*P* < 0.001)), had lower ASA (grades III–V: 30 *versus* 51 per cent (*P* < 0.001)), and were more likely to have surgery for benign conditions (71 *versus* 60 per cent; *P* < 0.001). There were no significant differences in the rates of emergency surgery, major surgery, or timing of SARS-CoV-2 infection between the groups. Of the 2391 patients, 759 (31.7 per cent) developed PPC. The 30-day mortality was 11.1 per cent (*n* = 265). Although rates of PPC were significantly lower in patients who had previously been vaccinated against BCG than those who did not (29 *versus* 38 per cent; *P* < 0.001) (*Table 1*), this did not persist on adjusted analysis (odds ratio (OR) 0.98, 95 per cent c.i. 0.79–1.22; *P* = 0.3) (*Table 1*). There were no significant differences in 30-day mortality rates in previously BCG-vaccinated patients *versus* unvaccinated patients (10 *versus* 13 per cent; *P* = 0.062), which remained on adjusted analysis (OR 1.25, 95 per cent c.i. 0.92–1.69; *P* = 0.156) (*Table 1*). Full models are presented in *Tables S2* and *S3*.

**Table 1:** Impact of BCG vaccination on postoperative pulmonary complications and 30-day mortality in patients with SARS-CoV-2 infection undergoing surgery

	No. of patients (%)	Unadjusted OR (95% c.i.)	*P*	Adjusted OR (95% c.i.)	*P*
**Pulmonary complications**					
No BCG	278 (37.5)	REF		REF	
BCG	481 (29.2)	0.69 (0.57–0.83)	0.001	0.98 (0.79–1.22)	0.878
**30-day mortality**					
No BCG	96 (12.9)	REF		REF	
BCG	169 (10.2)	0.77 (0.59–1.01)	0.053	1.25 (0.92–1.69)	0.156

OR, odds ratio; BCG, Bacillus Calmette-Guérin; REF, referent.

Stratified analysis performed by timing of vaccination demonstrated that patients who were vaccinated 15 years previously or longer were less likely than patients vaccinated less than 15 years ago to be ASA grades III–V (30 *versus* 41 per cent; *P* = 0.033) and were more likely to have surgery for benign disease (72 *versus* 57 per cent; *P* < 0.001) (*Table S4*). There were no significant differences in rates of postoperative pulmonary complications (29 *versus* 34 per cent; *P* = 0.208) or 30-day mortality (10 *versus* 14 per cent; *P* = 0.221), on adjusted analysis (*Tables S5* and *S6*).

There were important limitations to the present study. There was a broad inclusion of patients from low-, middle-, and high-income countries with varying pre- and postoperative procedures and availability of resources such as a well-equipped ICU for the management of the patient. Mortality and morbidity could have been greatly impacted by this difference. Some countries had a much higher prevalence and severity of disease, which could have also influenced outcomes.

According to these findings, prior BCG vaccination does not appear to protect SARS-CoV-2-infected patients against PPCs and 30-day mortality.

## Collaborators

Stephen Tabiri, Sivesh K Kamarajah, Dmitri Nepogodiev, Elizabeth Li, Joana Simoes, Sanskrithi Sravanam, Sheila Agyeiwaa Owusu, Haruna Mahama, Yaa Nyarko Agyeman, Joshua Arthur, Sheba Mary Kunfah, Frank Enoch Gyamfi, Emmanuel Abem Owusu, Markus W. Loffler, Paul Wandoh, Aneel Bhangu, Kwabena Siaw-Acheampong, Leah Argus, Daoud Chaudhry, Brett E Dawson, James C Glasbey, Rohan R Gujjuri, Conor S Jones, Sivesh K Kamarajah, Chetan Khatri, James M Keatley, Samuel Lawday, Elizabeth Li, Harvinder Mann, Ella J Marson, Kenneth A Mclean, Maria Picciochi, Elliott H Taylor, Abhinav Tiwari, Joana FF Simoes, Isobel M Trout, Mary L Venn, Richard JW Wilkin, Aneel Bhangu, Dmitri Nepogodiev, Irida Dajti (Albania), Arben Gjata (Albania), Luis Boccalatte (Argentina), Maria Marta Modolo (Argentina), Daniel Cox (Australia), Peter Pockney (Australia), Philip Townend (Australia), Felix Aigner (Austria), Irmgard Kronberger (Austria), Kamral Hossain (Bangladesh), Gabrielle VanRamshorst (Belgium), Ismail Lawani (Benin), Gustavo Ataide (Brazil), Glauco Baiocchi (Brazil), Igor Buarque (Brazil), Muhammad Gohar (Bulgaria), Mihail Slavchev (Bulgaria), Arnav Agarwal (Canada), Amanpreet Brar (Canada), Janet Martin (Canada), Maria Marta Modolo (Chile), Maricarmen Olivos (Chile), Jose Calvache (Colombia), Carlos Jose Perez Rivera (Colombia), Ana Danic Hadzibegovic (Croatia), Tomislav Kopjar (Croatia), Jakov Mihanovic (Croatia), Jaroslav Klat (Czech Republic), René Novysedlak (Czech Republic), Peter Christensen (Denmark), Alaa El-Hussuna (Denmark), Sylvia Batista (Dominican Republic), Eddy Lincango (Ecuador), Sameh H Emile (Egypt), Mengistu Gebreyohanes Mengesha (Ethiopia), Dr.Samuel Hailu (Ethiopia), Hailu Tamiru (Ethiopia), Joonas Kauppila (Finland), Alexis Arnaud (France), Markus Albertsmeiers (Germany), Hans Lederhuber (Germany), Markus Loffler (Germany), Stephen Tabiri (Ghana), Symeon Metallidis (Greece), Georgios Tsoulfas (Greece), Maria Aguilera Lorena (Guatemala), Gustavo Grecinos (Guatemala), Tamas Mersich (Hungary), Daniel Wettstein (Hungary), Dhruv Ghosh (India), Gabriele Kembuan (Indonesia), Peiman Brouk (Iran), Mohammad Khosravi (Iran), Masoud Mozafari (Iran), Ahmed Adil (Iraq), Helen M Mohan (Ireland), Oded Zmora (Israel), Marco Fiore (Italy), Gaetano Gallo (Italy), Francesco Pata (Italy), Gianluca Pellino (Italy), Sohei Satoi (Japan), Faris Ayasra (Jordan), Mohammad Chaar (Jordan), Ildar R Fakhradiyev (Kazakhstan), Mohammad Jamal (Kuwait), Muhammed Elhadi (Libya), Aiste Gulla (Lithuania), April Roslani (Malaysia), Laura Martinez (Mexico), Antonio Ramos De La Medina (Mexico), Oumaima Outani (Morocco), Pascal Jonker (Netherlands), Schelto Kruijff (Netherlands), Milou Noltes (Netherlands), Pieter Steinkamp (Netherlands), Willemijn van der Plas (Netherlands), Adesoji Ademuyiwa (Nigeria), Babatunde Osinaike (Nigeria), Justina Seyi-olajide (Nigeria), Emmanuel Williams (Nigeria), Sofija Pejkova (North Macedonia), Knut Magne Augestad (Norway), Kjetil Soreide (Norway), Zainab Al Balushi (Oman), Ahmad Qureshi (Pakistan), Raza Sayyed (Pakistan), Mustafa Abu Mohsen Daraghmeh (Palestine), Sadi Abukhalaf (Palestine), Moises Cukier (Panama), Hugo Gomez (Paraguay), Sebastian Shu (Peru), Ximena Vasquez (Peru), Marie Dione Parreno-Sacdalan (Philippines), Piotr Major (Poland), José Azevedo (Portugal), Miguel Cunha (Portugal), Irene Santos (Portugal), Ahmad Zarour (Qatar), Eduard-Alexandru Bonci (Romania), Ionut Negoi (Romania), Sergey Efetov (Russia), Andrey Litvin (Russia), Faustin Ntirenganya (Rwanda), Ehab AlAmeer (Saudi Arabia), Dejan Radenkovic (Serbia), Frederick Koh Hong Xiang (Singapore), Chew Min Hoe (Singapore), James Ngu Chi Yong (Singapore), Rachel Moore (South Africa), Ncamsile Nhlabathi (South Africa), Ruth Blanco Colino (Spain), Ana Minaya Bravo (Spain), Ana Minaya-Bravo (Spain), Umesh Jayarajah (Sri Lanka), Dakshitha Wickramasinghe (Sri Lanka), Mohammed Elmujtaba (Sudan), William Jebril (Sweden), Martin Rutegård (Sweden), Malin Sund (Sweden), Arda Isik (Turkey), Sezai Leventoğlu (Turkey), Tom EF Abbott (UK), Ruth Benson (UK), Ed Caruna (UK), Sohini Chakrabortee (UK), Andreas Demetriades (UK), Anant Desai (UK), Thomas D Drake (UK), John G Edwards (UK), Jonathan P Evans (UK), Samuel Ford (UK), Christina Fotopoulou (UK), Ewen Griffiths (UK), Peter Hutchinson (UK), Michael D Jenkinson (UK), Tabassum Khan (UK), Stephen Knight (UK), Angelos Kolias (UK), Elaine Leung (UK), Siobhan McKay (UK), Lisa Norman (UK), Riinu Ots (UK), Vidya Raghavan (UK), Keith Roberts (UK), Andrew Schache (UK), Richard Shaw (UK), Katie Shaw (UK), Neil Smart (UK), Grant Stewart (UK), Sudha Sundar (UK), Dale Vimalchandran (UK), Naomi Wright (UK), Sattar Alshryda (United Arab Emirates), Osaid Alser (United States), Kerry Breen (United States), Ian Ganly (United States), Haytham Kaafarani (United States), Brittany Kendall (United States), Hassan Mashbari (United States), Hamza Al Naggar (Yemen), Dennis Mazingi (Zimbabwe), EuroSurg, European Society of Coloproctology (ESCP), GlobalSurg, GlobalPaedSurg, ItSURG, PTSurg, SpainSurg, Italian Society of Colorectal Surgery (SICCR), Association of Surgeons in Training (ASiT), Irish Surgical Research Collaborative (ISRC), Joana FF Simoes (Chair), Dajti I (University Hospital Center Nene Tereza, Albania); Valenzuela JI (Hospital Velez Sarsfield, Argentina); Boccalatte LA, Gemelli NA, Smith DE (Hospital Italiano De Buenos Aires, Argentina); Dudi-Venkata NN, Kroon HM, Sammour T (Royal Adelaide Hospital, Australia); Roberts M, Mitchell D, Lah K, Pearce A, Morton A (Royal Brisbane and Women's Hospital, Australia); Dawson AC, Drane A (Gosford Hospital, Australia); Sharpin C, Nataraja RM, Pacilli M (Monash Childrens Hospital, Australia); Cox DRA, Muralidharan V, Riddiough GE, Clarke EM, Jamel W, Qin KR (Austin Hospital, Australia); Pockney P, Cope D, Egoroff N, Lott N (John Hunter Hospital, Australia); Putnis S, De Robles S, Ang Z (Wollongong Public Hospital, Wollongong, Australia); Mitteregger M, Uranitsch S, Stiegler M, Seitinger G, Aigner F (Krankenhaus der Barmherzigen Brüder Graz, Austria); Lumenta DB, Nischwitz SP, Richtig E, Pau M, Srekl-Filzmaier P, Eibinger N, Michelitsch B, Fediuk M, Papinutti A, Seidel G, Kahn J, Cohnert TU (Medical University of Graz, Austria); Messner F, Öfner D (Medical University of Innsbruck, Austria); Presl J, Varga M, Weitzendorfer M, Emmanuel K (Paracelsus Medical University, Salzburg, Austria); Binder AD (Universitätsklinikum Tulln, Tulln, Austria); Zimmermann M, Holawe S, Nkenke E, Grimm C, Kranawetter M (Medical University of Vienna, Vienna, Austria); Rahman Mitul A, Islam N, Karim S (Dhaka Shishu (Children) Hospital, Bangladesh); Komen N, Putnis S, De Robles S, Ang E (University Hospital Antwerp (UZA), Edegem, Belgium); De Praetere H, Tollens T, Schols G, Smets C, Haenen L (Imelda Hospital, Bonheiden, Belgium); Quintens J, Van Belle K (Europe Hospitals, Belgium); Van Ramshorst GH, Pattyn P, Desender L, Martens T, Van de putte D (Ghent University Hospital, Belgium); Lerut P, Grimonprez A, Janssen M, De Smul G, Wallaert P (AZ Groeninge, Belgium); Van den Eynde J, Oosterlinck W, Van den Eynde R, Sermon A, Boeckxstaens A, Cordonnier A, De Coster J, Jaekers J, Politis C, Miserez M (UZ Leuven, Belgium); Duchateau N, De Gheldere C (Heilig Hart Ziekenhuis Lier, Belgium); Flamey N, Pattyn P (AZ Delta, Belgium); Christiano A, Guidi B, Minussi AL, Castro S, Okoba W, Maldonado FHR, Oliveira P, Baldasso T, Santos L (Fundação Centro Médico De Campinas, Brazil); Gomes GMA, Buarque IL, Pol-Fachin L, Bezerra TS, Barros AV, da Silva AMR, Leite ALS, Silvestre DWA, Ferro CC (Hospital Santa Casa De Misericórdia De Maceió, Brazil); Araujo MS, Lopes LM, Damasceno PD, Araujo DHS (Hospital Universitário Antonio Pedro, Brazil); Laporte G, Salem MC (Irmandade Da Santa Casa De Misericórdia De Porto Alegre, Brazil); Guimaraes-Filho, MAC (Pedro Ernesto University Hospital, Brazil); Nacif L (Hospital Nove De Julho, Brazil); Flumignan RLG, Nakano LCU, Kuramoto DAB, Aidar ALS, Pereda MR, Correia RM, Santos BC, Carvalho AA, Amorim JE, Guedes Neto HJ, Areias LL, Sousa AF, Flumignan CDQ, Lustre WG, Moreno DH, Barros-Jr N, Baptista-Silva JCC (Hospital São Paulo, Brazil); Matos LL, Kowaski LP, Kulcsar MAV, Nunes KS, Teixeira MF (Instituto do Câncer do Estado de São Paulo, São Paulo, Brazil); Nunes RL, Ijichi TR, Kim NJ, Marreiro A, Muller B, Barakat Awada J (Notre Dame Intermédica - Hospital Salvalus, Brazil); Baiocchi G, Kowalski LP, Vartanian JG, Makdissi FB, Aguiar Jr S, Marques N, Carvalho GB, Marques TMDM, Abdallah EA, Zurstrassen CE, Gross JL, Zequi SC, Gonçalves BT, Santos SS, Duprat JP, Coimbra FJF (A.C. Camargo Cancer Center, Brazil); Cicco R (Instituto de Câncer Dr Arnaldo Vieira de Carvalho, São Paulo, Brazil); Takeda F, Cecconello I, Ribeiro Junior U (Instituto Do Câncer De Estado De São Paulo, Brazil); Gatti A, Oliva R, Nardi C (Hospital Geral De Pirajussara, Brazil); Slavchev M, Atanasov B, Belev N (University Hospital Eurohospital, Plovdiv, Bulgaria); Dell A, Bigam D, Dajani K, Al Riyami S (University of Alberta Hospital, Canada); Martin J, Cheng D, Yang H, Fayad A (London Health Sciences Centre and St Josephs Health Care London, Canada); Carrier FM, Amzallag E, Desroches J, Ruel M (Centre hospitalier de l’Université de Montréal, Canada); Caminsky NG, Boutros M, Moon J, Wong EG, Vanounou T, Pelletier J, Wong S, Girsowicz E, Bayne J, Obrand D, Gill H, Steinmetz O, MacKenzie K, Lukaszewski M, Jamjoum G (Jewish General Hospital, Canada); Richebé P, Verdonck O, Discepola S, Godin N, Idrissi M (Hôpital Maisonneuve-Rosemont, Canada); Briatico D, Sharma S, Talwar G, Bailey K (McMaster Children’s Hospital, Hamilton, Ontario, Canada); Lecluyse V (Hôpital du Sacré-Cœur-de-Montréal, Canada); Côté G (Centre hospitalier universitaire Sainte-Justine, Canada); Demyttenaere S, Garfinkle R, Boutros M (St. Mary's Hospital, Canada); Kouyoumdjian A, Boutros M, Dumitra S, Moon J, Wong EG, Khwaja K, Luo L, Lukaszewski M, Gill H, Berry G, Liberman AS, Girsowicz E, Bayne J, Obrand D, Steinmetz O, MacKenzie K, Schmid S, Spicer J, Al Farsi M (McGill University Health Center, Canada); Abou-Khalil J (The Ottawa Hospital, Canada); Couture E, Mohammadi S, Tremblay H, Gagné N, Bergeron A (Institut universitaire de cardiologie et de pneumologie de Québec, Canada); Turgeon Af, Costerousse O, Bellemare D, Babin C, Blier C (Centre hospitalier universitaire de Québec, Canada); Wood ML, Persad A, Groot G, Pham H (Saskatoon City Hospital/Royal University Hospital/St. Paul's Hospital, Canada); D'Aragon F, Carbonneau E, Bouchard M, Masse M, Pesant F, Héroux J (Centre hospitalier universitaire de Sherbrooke, Canada); Karanicolas P, Hallet J, Nadler A, Nathens A (Sunnybrook Hospital, Canada); Ko M, Brar A (St. Joseph's Health Centre, Canada); Mayson K (Vancouver General Hospital, Canada); Kidane B, Srinathan S (Health Sciences Centre, Canada); Escudero MI, Reyes JT (Hospital Clinico Universidad De Chile/ Hospital San Jose, Chile); Modolo MM, Ramirez Nieto P, Sepulveda R, Bolbarán A, Molero A, Ruiz I (Barros Luco Trudeau, Chile); Reyes GP, Salas R, Suazo C (Hospital del Salvador, Chile); Muñoz R, Grasset E, Inzunza M, Besser N, Irarrázaval MJ, Jarry C, Bellolio F, Romero Manqui CA, Ruiz Esquide M (Hospital Clínico Universidad Católica, Chile); Fuentes T, Campos J (Hospital Sotero Del Rio, Chile); Perez Rivera CJ, Cabrera PA (Fundacion Cardioinfantil-Instituto de Cardiología, Colombia); Pinilla RE, Guevara O, Jimenez Ramirez LJ, Velasquez Cuasquen BG, Herrera Mora DR, Bonilla A, Diaz S, Manrique E, Facundo H, Velez Bernal JL, Ángel J, García M, Guzmán L, Lehmann C, Cervera S, Trujillo Sanchez LM (Instituto Nacional De Cancerologia, Colombia); Guevara R, Valbuena D, Suarez L, Jimenez G, Velandia A, Vargas J, Espinosa J, Rey S (Clínica Universitaria Colombia, Bogotá., Colombia); Mendoza Quevedo Jairo (Subred Sur Occidente de Kennedy (Hospital de Kennedy), Bogota, Colombia); Calvache JA, Orozco-Chamorro CM, Sánchez-Gómez TA, Rojas-Tejada DA (Hospital Universitario San José, Popayán, Colombia); Mihanovic J, Bakmaz B, Rakvin I, Sulen N, Andabaka T (Zadar General Hospital, Croatia); Luksic I, Mamic M (University Hospital Dubrava, Zagreb, Croatia); Martinek L, Skrovina M (Hospital & Oncological Centre Novy Jicin, Czechia); Žatecký J, Peteja M (Slezská nemocnice v Opavě, p.o., Czechia); Kristensen HØ, Mekhael M, Christensen P, Westh L (Aarhus University Hospital, Denmark); Smith H, Haugstvedt AF, Jönsson ML (Bispebjerg Hospital, Denmark); Crespo A, Batista S, Rodriguez-Abreu J, Tactuk N, Diaz-Delgado PJ, Rivas R (Cedimat - Centro De Diagnóstico, Medicina Avanzada, Laboratorio Y Telemedicina, Dominican Republic); Sarmiento-Bobadilla JA (Hospital General Norte de Guayaquil, Guayaquil, Ecuador); Ashoush F, Samir Abdelaal A, Qatora MS, Elsayed Hewalla ME, Metwalli M, Atta R, Abdelmajeed A, Abosamak NE, Sabry A, Shehata S (Alexandria Main University Hospital, Egypt); Sallam I, Amira G, Sherief M, Sherif A (Misr Cancer Center, Egypt); Salem H, Hamdy R, Aboulkassem H, Ghaly G, Sherif G, Morsi A, Abdelrahman A (National Cancer Institute, Egypt); Omnia Ahmed (Coptic Hospital, Cairo, Egypt); Tawheed A, El Kassas M, Omar W (Endemic Medicine Department, Faculty of Medicine, Helwan University, Egypt); Abdelsamed A, Seleim A, Salem H, Azzam AY (Al Azhar University Hospitals, Egypt); ElFiky M, Nabil A (Kasr Alainy Faculty of Medicine, Cairo University, Egypt); Ibraheem M, Ghaly G, ELDeeb M, Fawzy M (Baheya Center for Early detection and Treatment of Breast Cancer, Egypt); Hamed H (Elkheir Hospital, Egypt); Emile S, Elfallal A, Elfeki H, Shalaby M, Sakr A (Mansoura University Hospital, Egypt); Alrahawy M, Atif H, Sakr A, Soltan H (Menofiya University Hospital, Egypt); Sayed AK, Salah A, Atiya A (Minia University Hospital, Egypt); Wassim K (Quwaysna Central Hospital, Egypt); Abbas AM, Abd Elazeem HAS, Abd-Elkariem AY, Abd-Elkarem MM, Alaa S, Ali AK, Ashraf M, Ayman A, Azizeldine MG, Elkhayat H, Emad Mashhour A, Gaber M, Hamza HM, Hawal I, Hetta HF, Elghazaly SM, Mohammed MM, Monib FA, Nageh MA, Saad A, Saad MM, Shahine M, Yousof EA, Youssef A (Assiut University Hospital, Assiut, Egypt); Esmail E, Khalaf M (Kafr Elzyat Hospital, Egypt); Eldaly A (El-Menshawy Hospital, Egypt); Ghoneim A, Hawila A, Badr H, Elhalaby I, Abdel-bari M, Elbahnasawy M, Hamada MK, Morsy MS, Hammad M, Hammad M, Essa M, Fayed MT, Elzoghby M, Rady M, Hamad O, Salman S, Sarsik S, Abd-elsalam S, Gamal Badr S, El-Masry Y (Tanta University Hospital, Egypt); Moahmmed MMH (Zagazig University Hospitals, Zagazig, Egypt); Hailu S, Wolde A, Mengesha M, Nida S, Workneh M, Y Ahmed M, Fisseha T, Kassa D, Zeleke H, Admasu A, Laeke T, Tirsit A, Gessesse M, Addissie A (Tikur Anbessa (Black Lion) Hospital-Addis Ababa University, Ethiopia); Bekele K (Maddawalabu University Goba Referral Hospital, Ethiopia); Kauppila JH, Sarjanoja E (Länsi-Pohja Central Hospital, Finland); Testelin S, Dakpé S, Devauchelle B, Bettoni J, Lavagen N (CHU Amiens, France); Schmitt F, Lemée JM, Boucher S, Breheret R, Kün-Darbois JD, Kahn A, Gueutier A, Bigot P (CHU Angers, France); Borraccino B (Centre Hospitalier Auxerre, Auxerre, France); Lakkis Z, Doussot A, Heyd B, Manfredelli S, Mathieu P, Paquette B, Turco C, Barrabe A, Louvrier A (CHU Besançon, France); Moszkowicz D, Giovinazzo D, Bretagnol F (Hôpital Louis-Mourier - Ap-Hp, France); Police A, Charre L, Volpin E, Braham H, El Arbi N, Villefranque V, Bendjemar L (Hôpital Simone Veil, France); Girard E, Abba J, Trilling B (CHU Grenoble-Alpes, Hôpital Michallon, France); Chebaro A, Lecolle K, Truant S, El Amrani M, Zerbib P, Pruvot FR, Mathieu D, Surmei E, Mattei L, Marin H (CHU Lille, France); Christou N, Ballouhey Q, Ferrero P, Coste Mazeau P, Tricard J, Barrat B, Taibi A, Usseglio J, Laloze J, Salle H, Fourcade L (Chu Limoges, France); Duchalais E, Regenet N, Rigaud J, Waast D, Denis W, Malard O, Buffenoir K, Espitalier F, Ferron C, Varenne Y, Crenn V, De Vergie S, Cristini J, Samarut E (Nantes University Hospital, France); Tzedakis S, Bouche PA, Gaujoux S (Hôpital Cochin - Aphp, France); Kantor E (AP-HP Hopital Bichat Claude Bernard, Paris, France); Gossot D, Seguin-Givelet A, Fuks D, Grigoroiu M, Sanchez Salas R, Cathelineau X, Macek P, Barbé Y, Rozet F, Barret E, Mombet A, Cathala N, Brian E, Zadegan F, Conso C (Institut Mutualiste Montsouris, France); Blanc T, Broch A, Sarnacki S, (Necker Enfants Malades University Children Hospital - APHP, Paris; Université de Paris, Paris, France); Ali L, Bonnard A, Peycelon M (Robert-Debré Children University Hospital - APHP, Paris; Université de Paris, Paris, France); Hervieux E, Clermidi P, Maisonneuve E, Aubry E, Thomin A, Langlais T (Hôpital Trousseau - Aphp, France); Passot G, Glehen O, Cotte E, Lifante JC (Hopital Lyon Sud, Pierre Bénite, France); De Simone B, Chouillard E (Centre Hospitalier Intercommunal Poissy Saint Germain En Laye, France); Arnaud AP, Violas P (CHU Rennes - Hôpital Sud, France); Bergeat D, Merdrignac A, Arnaud AP (Chu Rennes - Hopital Pontchaillou, France); Scalabre A, Perotto LO, Le Roy B, Haddad E, Vermersch S (Chu Saint Etienne, France); Ezanno AC, Barbier O, Vigouroux F, Malgras B, Aime A (Hia Begin, France); Seeliger B, Mutter D, Philouze G, Pessaux P (IHU-Strasbourg / Strasbourg University Hospitals, France); Germain A, Chanty H, Ayav A (CHRU Nancy, France); Kassir R, Von theobald P, Sauvat F (CHU Reunion, France); O'Connor J, Mayombo Idiata M, O'Connor Z, Tchoba S (Bongolo Hospital, Gabon); Modabber A, Winnand P, Hölzle F (University Hospital Aachen, Germany); Sommer B, Shiban E, Wolf S, Anthuber M, Sommer F (University Hospital Augsburg, Germany); Kaemmerer D, Schreiber T (Zentralklinik Bad Berka, Germany); Kamphues C, Lauscher JC, Schineis C, Loch FN, Beyer K (Charité University Medicine, Campus Benjamin Franklin, Germany); Nasser S, Sehouli J (Charité Comprehensive Cancer Center, Germany); Höhn P, Braumann C, Reinkemeier F, Uhl W (St Josef-Hospital, Bochum, Germany); Weitz J, Bork U, Welsch T, Praetorius C, Korn S, Distler M (University Hospital Carl Gustav Carus, Technical University Dresden, Germany); Fluegen G, Knoefel WT, Vay C (University Hospital Duesseldorf, Germany); Golcher H, Grützmann R, Binder J (Universitätsklinikum Erlangen, Germany); Meister P, Gallinat A, Paul A (University Hospital Essen, Essen, Germany); Schnitzbauer AA, Thoenissen P, El Youzouri H, Schreckenbach T, Nguyen TA (Frankfurt University Hospital, Goethe University, Frankfurt, Germany); Eberbach H, Bayer J, Erdle B, Sandkamp R (Medical Center – Albert-Ludwigs-University of Freiburg, Faculty of Medicine, Germany); Nitschke C, Izbicki J, Uzunoglu FG, Koenig D, Gosau M, Böttcher A, Heuer A, Klatte TO, Priemel M, Betz CS, Burg S, Möckelmann N, Busch CJ, Bewarder J, Zeller N, Smeets R, Thole S, Vollkommer T, Speth U, Stangenberg M (University Medical Center Hamburg-Eppendorf, Germany); Hakami I, Boeker C, Mall J (KRH Nordstadt-Siloah Hospitals, Hannover, Germany); Schardey HM, von Ahnen T, von Ahnen M, Brunner U (Agatharied Hospital, Hausham, Germany); Tapking C, Kneser U, Hirche C, Jung M, Kowalewski KF (BG Trauma Center Ludwigshafen, Germany); Kienle P (Theresienkrankenhaus, Mannheim, Germany); Reissfelder C, Seyfried S, Herrle F, Hardt J, Galata C, Birgin E, Rahbari N, Vassos N (Mannheim University Medical Center (Universitätsmedizin Mannheim), Germany); Stoleriu MG, Hatz R (Asklepios Pulmonary Hospital, Munich Gauting, Germany); Albertsmeier M, Börner N, Lampert C, Werner J (Department of General, Visceral and Transplantation Surgery, Ludwig-Maximilians-Universität Munich, Munich, Germany); Kuehlmann B, Prantl L (University Hospital Regensburg, Regensburg, Germany); Brunner SM, Schlitt HJ, Brennfleck F, Pfister K, Oikonomou K (University Medical Center Regensburg, Department of Surgery, Germany); Reinhard T, Nowak K (RoMed Klinikum Rosenheim, Germany); Ronellenfitsch U, Kleeff J, Delank KS, Michalski CW, Szabo G (University Hospital Halle (Saale), Germany); Widyaningsih R, Stavrou GA (Klinikum Saarbrücken, Germany); Bschorer R, Mielke J, Peschel T (Helios Kliniken Schwerin, Germany); Königsrainer A, Quante M, Löffler MW, Yurttas C (University Hospital Tübingen, Department of General, Visceral and Transplant Surgery, Germany); Doerner J, Seiberth R (Helios Universitätsklinikum Wuppertal (Universität Witten/Herdecke), Germany); Bouchagier K, Klimopoulos S, Paspaliari D, Stylianidis G (Evaggelismos General Hospital, Greece); Syllaios A, Baili E, Schizas D, Liakakos T, Charalabopoulos A, Zografos C, Spartalis E (Laiko University Hospital, Greece); Manatakis DK, Tasis N, Antonopoulou MI (Athens Naval and Veterans Hospital, Greece); Xenaki S, Xynos E, Chrysos E, Athanasakis E, Tsiaousis J (University Hospital of Heraklion Crete and Interclinic Hospital of Crete, Greece); Lostoridis E, Tourountzi P (Kavala General Hospital, Greece); Tzovaras G, Tepetes K, Zacharoulis D, Baloyiannis I, Perivoliotis K, Hajiioannou J, Korais C, Gkrinia E, Skoulakis CE, Saratziotis A, Koukoura O, Symeonidis D, Diamantis A (General University Hospital of Larissa, Greece); Tsoulfas G, Christou CD, Tooulias A, Papadopoulos V, Anthoulakis C, Grimbizis G, Zouzoulas D, Tsolakidis D (Papageorgiou General Hospital, Greece); Tatsis D, Christidis P, Loutzidou L, Ioannidis O, Astreidis I, Antoniou A, Antoniadis K, Vachtsevanos K, Paraskevopoulos K, Kalaitsidou I, Alexoudi V, Stavroglou A, Mantevas A, Michailidou D, Grivas T, Deligiannidis D, Politis S, (George Papanikolaou General Hospital of Thessaloniki, Greece); Barrios Duarte A, Portilla AL, Lowey MJ, Recinos G, Lopez Muralles I (Hospital General De Enfermedades, Guatemala); Siguantay MA (Hospital Roosevelt, Guatemala City, Guatemala); Estrada EE, Aguilera-Arévalo ML, Cojulun JM, Echeverría-Dávila G (Hospital General San Juan De Dios, Guatemala City, Guatemala); Marín C, Icaza de Marín GC (Hospital Regional De Zacapa, Guatemala); Kok SY, Joeng HKM, Chan LL, Lim D (United Christian Hospital, Hong Kong); Novak Z, Echim T (National Institute of Oncology, Hungary); Suszták N, Banky B (Szent Borbála Kórház, Hungary); Kembuan G, Pajan H, Islam AA (Rsud Wahidin Sudirohusodo, Indonesia); Rahim F (Baghaei Hospital, Iran); Safari H (Golestan Hospital & Ahvaz Jundishapur University of Medical Sciences, Iran); Mozafari M, Brouki Milan P, Tizmaghz A, Rezaei Tavirani M (Firoozabadi Hospital, Iran); Ahmed A (Baghdad Medical City, Iraq); Hussein R (Zafaraniyah General Hospital, Iraq); Fleming C, O'Brien S, Kayyal MY, Daly A, Killeen S, Corrigan M (Cork University Hospital, Ireland); De Marchi J, Hill A, Farrell T, Davis NF, Kearney D, Nelson T (Beaumont Hospital, Ireland); Maguire PJ, Barry C, Farrell R, Smith LA, Mohan HM, Mehigan BJ, Mccormick P, Larkin JO, Fahey BA, Rogers A, Donlon N, O'Sullivan H, Nugent T, Reynolds JV, Donohue C, Shokuhi P, Ravi N, Fitzgerald C, Lennon P, Timon C, Kinsella J, Smith J, Boyle T, Alazawi D, Connolly E, Butt W, Croghan SM, Manecksha RP (St. James's Hospital, Ireland); Fearon N, Winter D, Heneghan H, Maguire D, Gallagher T, Conlon K, Kennedy N, Martin S, Kennelly R, Hanly A, Ng KC, Fagan J, Geary E, Cullinane C, Hanly A, Carrington E, Geraghty J, McDermott E, Pritchard R, McPartland D, Boland M, Stafford A, Maguire D, Geoghegan J (St Vincent's University Hospital, Ireland); Elliott JA, Ridgway PF, Gillis AE, Bass GA, Neary PC, O'Riordan JM, Kavanagh DO, Reynolds IS, Conlon K, Joyce DP, Boyle E, Egan B, Whelan M, Elkady R, Tierney S, Connelly TM, Earley H, Umair M, O'Connell C, Manecksha RP, Thomas AZ, Rice D, Madden A, Bashir Y, Creavin B (Tallaght Hospital, Ireland); Cullivan O, Owens P, Canas-Martinez A, Murphy C, Pickett L, Murphy B, Mastrosimone A, Beddy D, Arumugasamy M, Allen M, Aremu M (Connolly Hospital Blanchardstown, Ireland); McCarthy C, O’Connor C, O'Connor DB, Kent E, Malone F, Geary M (Rotunda Hospital, Ireland); McKevitt KL, Lowery AJ, Ryan ÉJ, Aherne TM, Fowler A, Hassanin A, Hogan AM, Collins CG, Finnegan L, Carroll PA, Kerin MJ, Walsh SR (University Hospital Galway, Ireland); Nally D, Peirce C, Coffey JC, Cunningham RM, Tormey S (University Hospital Limerick, Ireland); Hardy NP, Neary PM (University Hospital Waterford/University College Cork, Ireland); Muallem-Kalmovich L, Kugler N, Lavy R, Zmora O (Shamir Medical Center, Israel); Horesh N (Sheba Medical Center, Israel); Vergari R (Ospedali Riuniti Di Ancona, Italy); Mochet S, Barmasse R, Usai A, Morelli L (Ospedale Regionale Umberto Parini, Italy); Picciariello A, Papagni V, Altomare DF (Azienda Ospedaliero Universitaria Consorziale Policlinico Di Bari, Italy); Colledan M, Zambelli MF, Tornese S, Camillo A, Rausa E, Bianco F, Lucianetti A (Asst-Papa Giovanni Xxiii- Bergamo, Italy); Prucher GM, Baietti AM, Ruggiero F, Maremonti P, Neri F, Ricci S, Biasini M, Zarabini AG (Ospedale Maggiore/Bellaria Carlo Alberto Pizzardi Ausl Bologna, Italy); Belvedere A, Bernante P, Bertoglio P, Boussedra S, Brunocilla E, Cipriani R, Cisternino G, De Crescenzo E, De Iaco P, Della Gatta AN, Dondi G, Frio F, Jovine E, Mineo Bianchi F, Neri J, Parlanti D, Perrone AM, Pezzuto AP, Pignatti M, Pilu G, Pinto V, Poggioli G, Ravaioli M, Rottoli M, Schiavina R, Serenari M, Serra M, Solli P, Taffurelli M, Tanzanu M, Tesei M, Violante T, Zanotti S (Sant'orsola Hospital, Alma Mater Studiorum University of Bologna, Italy, Italy); Tonini V, Sartarelli L, Cervellera M, Gori A (S.Orsola-Malpighi Hospital, Italy); Armatura G, Scotton G, Patauner S, Frena A (St. Moritz Hospital, Italy); Podda M, Pisanu A, Esposito G, Frongia F (Cagliari University Hospital, Italy); Abate E, Laface L, Casati M, Schiavo M, Casiraghi T (Ospedale Vittorio Emanuele III - Carate Brianza, Italy); Sammarco G, Gallo G, Vescio G, Fulginiti S, Scorcia V, Giannaccare G, Carnevali A (University ‘Magna Graecia’ of Catanzaro, Italy); Giuffrida MC, Marano A, Palagi S, Di Maria Grimaldi S, Testa V, Peluso C, Borghi F, Simonato A, Puppo A, D'Agruma M, Chiarpenello R, Pellegrino L, Maione F, Cianflocca D, Pruiti Ciarello V, Giraudo G, Gelarda E, Dalmasso E, Abrate A, Daniele A, Ciriello V, Rosato F, Garnero A, Leotta L (Santa Croce E Carle Hospital, Cuneo, Italy); Giacometti M, Zonta S (San Biagio Hospital, Domodossola - ASL VCO, Italy); Lomiento D, Taglietti L, Dester S, Compagnoni B, Viotti F, Cazzaniga R, Del Giudice R (Asst Valcamonica Ospedale Di Esine, Italy); Mazzotti F, Pasini F, Ugolini G (Ospedale per gli Infermi di Faenza, Italy); Fabbri N, Feo CV, Righini E, Gennari S (Azienda Unità Sanitaria Locale di Ferrara, Università di Ferrara, Italy); Chiozza M, Anania G, Urbani A, Koleva Radica M, Carcoforo P, Portinari M, Sibilla M (Azienda Ospedaliero Universitaria Sant'Anna, Italy); Anastasi A, Bartalucci B, Bellacci A, Canonico G, Capezzuoli L, Di Martino C, Ipponi P, Linari C, Montelatici M, Nelli T, Spagni G, Tirloni L, Vitali A (Ospedale San Giovanni Di Dio, Italy); Agostini C, Alemanno G, Bartolini I, Bergamini C, Bruscino A, Checcucci C, De Vincenti R, Di Bella A, Fambrini M, Fortuna L, Maltinti G, Muiesan P, Petraglia F, Prosperi P, Ringressi MN, Risaliti M, Sorbi F, Taddei A (Azienda Ospedaliera Universitaria Careggi, Italy); Lizzi V, Vovola F, Arminio A, Cotoia A, Sarni AL (Ospedali Riuniti Azienda Ospedaliera Universitaria Foggia, Italy); Familiari P, D'Andrea G, Picotti V, Bàmbina F (Fabrizio Spaziani, Italy); Fontana T (Vittorio Emanuele, Italy); Barra F, Ferrero S, Gustavino C, Kratochwila C, Ferraiolo A, Costantini S, Batistotti P, Aprile A, Almondo C, Ball L, Robba C, Scabini S, Pertile D, Massobrio A, Soriero D (IRCCS Ospedale Policlinico San Martino, Italy); D'Ugo S, Depalma N, Spampinato MG ("Vito Fazzi" Hospital, Italy); Lippa L, Gambacciani C, Santonocito OS, Aquila F, Pieri F (Spedali Riuniti Di Livorno, Italy); Ballabio M, Bisagni P, Longhi M, Armao T, Madonini M, Gagliano A, Pizzini P (Ospedale Maggiore Di Lodi, Italy); Costanzi A, Confalonieri M, Monteleone M, Colletti G, Frattaruolo C, Mari G (San Leopoldo Mandic, Italy); Spinelli A, Mercante G, Spriano G, Gaino F, Ferreli F, De Virgilio A, Rossi V, Carvello MM, Di Candido F, Kurihara H, Marrano E, Torzilli G, Castoro C, Carrano FM (Humanitas Clinical and Research Center - IRCCS, Rozzano (MI), Italy); Martinelli F, Macchi A, Fiore M, Pasquali S, Cioffi SPB, Baia M, Abatini C, Sarre C, Mosca A, Biasoni D, Gronchi A, Citterio D, Mazzaferro V, Cadenelli P, Gennaro M, Capizzi V, Guaglio M, Sorrentino L, Bogani G, Sarpietro G, Giannini L, Comini LV, Rolli L, Folli S, Raspagliesi F, Piazza C, Cosimelli M, Salvioni R (Fondazione IRCCS Istituto Nazionale dei Tumori, Milano, Italy); Antonelli B, Baldari L, Boni L, Cassinotti E, Pignataro L, Rossi G, Torretta S, Beltramini GA, Gianni' A (Fondazione IRCSS Ca' Granda - Ospedale Maggiore Policlinico, Italy); Tagliabue M, De Berardinis R, Pietrobon G, Chu F, Cenciarelli S, Adamoli L, Ansarin M, Fumagalli Romario U, Mastrilli F (Istituto Europeo Di Oncologia - IRCCS, Milano, Italy); Mariani NM, Nicastro V (Asst Santi Paolo E Carlo, Italy); Cellerino P (Ospedale Fatebenefratelli E Oftalmico, Italy); Colombo F, Frontali A, Bondurri A, Guerci C, Maffioli A, Ferrario L (Ospedale Luigi Sacco Milano, Italy); Candiani M, Bonavina G, Ottolina J, Valsecchi L, Mortini P, Gagliardi F, Piloni M, Medone M, Negri G, Bandiera A, De Nardi P, Sileri P, Carlucci M, Pelaggi D, Rosati R, Vignali A, Parise P, Elmore U (San Raffaele Scientific Institute, Milan, Italy); Tamini N, Nespoli LC, Rennis M, Pitoni L, Chiappetta MF, Vico E, Fruscio R, Grassi T (Ospedale San Gerardo, Università degli Studi di Milano Bicocca, Italy); Sasia D, Migliore M, Gattolin A, Rimonda R, Travaglio E, Olearo E (Regina Montis Regalis Hospital, Mondovì, Italy); Tufo A, Marra E, Maida P, Marte G, Tammaro P (Ospedale Del Mare, Italy); Bianco F, Incollingo P (Ospedale S. Leonardo - Asl Napoli 3 Sud, Castellammare Di Stabia, Italy); Izzo F, Belli A, Patrone R, Albino V, Leongito M, Granata V, Piccirillo M, Palaia R (Istituto Nazionale Tumori Fondazione, G. Pascale-IRCCS, Naples, Italy); Francone E, Gentilli S, Nikaj H (Azienda Ospedaliero Universitaria Maggiore Della Carità, Novara, Italy); Fiorini A, Norcini C, Chessa A (San Giovanni Di Dio, Italy); Marino MV, Mirabella A, Vaccarella G (Azienda Ospedaliera, Ospedali Riuniti Villa Sofia-Cervello, Palermo, Italy); Musini L, Ampollini L, Bergonzani M, Varazzani A, Bellanti L, Domenichini M, Rossi G, Cabrini E, Fornasari A, Freyrie A, Dejana DO, D'Angelo G, Bertoli G, Di Lella F, Bocchialini G, Falcioni M, Lanfranco D, Poli T (Azienda Ospedaliero-Universitaria di Parma, Italy); Giuffrida M, Annicchiarico A, Perrone G, Catena F (Parma University Hospital, Italy); Raffaele A (Policlinico San Matteo, Italy); De Manzoni Garberini A (Ospedale Civile Spirito Santo, Italy); Baldini E, Conti L, Ribolla M, Capelli P, Isolani SM, Maniscalco P, Cauteruccio M, Ciatti C, Puma Pagliarello C, Gattoni S (Ospedale “Guglielmo Da Saliceto” - Dipartimento di Chirurgia - Piacenza, Italy); Galleano R, Malerba M, Ciciliot M (Ospedale Santa Corona, Pietra Ligure (SV), Pietra Ligure, Italy); Farnesi F, Calabrò M, Pipitone Federico NS, Lunghi EG, Muratore A (Edoardo Agnelli, Italy); Morelli L, Di Franco G, Palmeri M, Tartaglia D, Coccolini F, Chiarugi M, Simoncini T, Gadducci A, Caretto M, Giannini A, Perutelli A, Domenici L, Garibaldi S, Capanna R, Andreani L, Furbetta N, Guadagni S, Bianchini M, Gianardi D (Azienda Ospedaliero Universitaria Pisana, Italy); Pinotti E, Montuori M, Carissimi F, Baronio G (Policlinico San Pietro, Italy); Zizzo M, Castro Ruiz C, Annessi V, Montella MT, Falco G, Mele S, Ferrari G, Mastrofilippo V, Mandato VD, Aguzzoli L (Azienda Unità Sanitaria Locale - IRCCS Di Reggio Emilia, Italy); Corbellini C, Baldi C, Sampietro GM (Ospedale Di Rho - Asst Rhodense, Italy); Palini GM, Zanini N, Garulli G (Ospedale Infermi di Rimini, Rimini, Italy); Barone R, Murgese A, Mungo S, Grasso M, Marafante C, Birolo SL, Moggia E, Caccetta M, Masciandaro A, Deirino A, Garino M (Ospedale Degli Infermi Di Rivoli, Italy); Perinotti R, Maiello F (Ospedale degli Infermi, Biella, Italy); Gordini L, Lombardi CP, Marzi F, Marra AA, Ratto C, Di Muro M, Litta F, De Simone V, Cozza V, Rosa F, Agnes A, Parello A, Alfieri S, Sganga G (Fondazione Policlinico Universitario Agostino Gemelli IRCCS, Italy); Lapolla P, Mingoli A, De Toma G, Fiori E, La Torre F, Sapienza P, Brachini G, Cirillo B, Iannone I, Zambon M, Chiappini A, Meneghini S, Fonsi GB, Cicerchia PM, Bruzzaniti P, Santoro A, Frati A, Marruzzo G, Ribuffo D (Policlinico Umberto I Sapienza University of Rome, Italy); Sagnotta A, Marino Cosentino L, Mancini S (Ospedale San Filippo Neri, Italy); Lisi G, Spoletini D (Sant'Eugenio Hospital, Italy); Bellato V Campanelli M, Sica G, Siragusa L, (Policlinico Tor Vergata Hospital, Rome, Italy); Bonavina L, Asti E, Bernardi D, Lovece A (IRCSS Policlinico San Donato, University of Milan, Italy); Perra T, Porcu A, Fancellu A, Feo CF, Scanu AM (Cliniche San Pietro, A.O.U. Sassari, Italy); Tuminello F, Galleano R, Franceschi A, Langone A (San Paolo, Italy); Fleres F, Spolini A, Bordoni P, Franzini M, Clarizia G, Grechi A, Longhini A (Ospedale Di Sondrio (Asst Valtellina E Alto Lario), Italy); Guaitoli E, Manca G (Perrino Hospital Brindisi, Italy); Grossi U, Novello S, Zanus G, Romano M, Rossi S (Ca' Foncello Treviso - DISCOG, Università di Padova, Italy); Ferrara F (San Carlo Borromeo Hospital, ASST Santi Paolo e Carlo, Italy); La Torre M (Fabia Mater Hospital, Rome, Italy); Pirozzolo G, Recordare A (Dell'Angelo Hospital, ULSS3 Serenissima, Venezia, Italy); Paiella S, Turri G, Rattizzato S, Campagnaro T, Guglielmi A, Pedrazzani C, Ruzzenente A, Poletto E, Conci S, Casetti L, Fontana M, Salvia R, Malleo G, Esposito A, Landoni L, De Pastena M, Bassi C, Tuveri M, Nobile S, Marchegiani G, Bortolasi L (Azienda Ospedaliera Universitaria Integrata Di Verona, Italy); Sambugaro E, Malavolta M, Moretto G, Impellizzeri H, Inama M (Ospedale Pederzoli, Italy); Barugola G, Ascari F, Ruffo G (IRCCS Ospedale Sacro Cuore Don Calabria, Negrar di Valpolicella (Verona), Italy); Granieri S, Cotsoglou C (Asst Vimercate, Italy); Berselli M, Desio M, Marchionini V, Cocozza E (ASST Settelaghi, Varese, Italy); Di Saverio S, Ietto G, Iovino D, Carcano G (University of Insubria, Ospedale di Circolo e Fondazione Macchi (Varese), Varese Lombardy, Italy); Ayasra F, Qasem A, Ayasra Y (Al-Basheer Hospital, Jordan); Al-Masri M, Abou Chaar MK, Al-Najjar H, Ghandour K, Alawneh F, Abdel Jalil R, Abdel Al S, Elayyan M, Ghanem R, Lataifeh I, Alsaraireh O (King Hussein Cancer Center, Jordan); Abu Za’nouneh FJ, Fahmawee T, Ibrahim A, Obeidat K (King Abdullah University Hospital, Jordan); Lee KJ (Keimyung University School of Medicine, Daegu, Korea); Shin SJ, Chung H (Keimyung University, Daegu, Korea); Albader I, Alabbad J, Albader MAS (Mubarak Al-Kabeer Hospital, Jabriya, Kuwait); Bouhuwaish A, Taher AS, Omar MSM (Tobruk Medical Center, Libya); Abdulwahed E, Biala M, Morgom M (Tripoli Central Hospital, Libya); Elhadi A, Alarabi A, Msherghi A, Elhajdawe F, Alsoufi A (Tripoli University Hospital, Libya); Salamah A, Salama H, Bulugma M, Almabrouk H (Zawia Teaching Hospital, Libya); Venskutonis D, Dainius E, Kubiliute E, Bradulskis S, Parseliunas A, Kutkevicius J, Subocius A (Lithuanian University of Health Sciences Kaunas Clinical Hospital, Lithuania); Cheong YJ, Masood MS (Hospital Raja Permaisuri Bainun, Ipoh, Malaysia); Ngo CW, Saravanan R, Abdul Maei N (Hospital Enche' Besar Hajjah Khalsom, Malaysia); Hayati F, Amin Sahid N (Queen Elizabeth Hospital & Universiti Malaysia Sabah, Kota Kinabalu, Sabah, Malaysia); Yanowsky Reyes G, Orozco Perez J, Damian R, Santana Ortiz R, Colunga Tinajero CA (Antiguo Hospital Civil De Guadalajara, Mexico); Cordera F, Gómez-Pedraza A, Maffuz-Aziz A, Posada JA, De la Rosa Abaroa MA, Alvarez MR, Arrangoiz R, Hernández R (ABC Medical Center, Mexico); Bozada Gutierrez K, Trejo-Avila M, Valenzuela-Salazar C, Herrera-Esquivel J, Moreno-Portillo M (Hospital General Dr. Manuel Gea González, Mexico); Pinto-Angulo VM, Sosa-Duran EE, Ziad-Aboharp H, Jimenez Villanueva X (Hospital Juárez de México, Ciudad de México, Mexico); Soulé Martínez CE, Lupián-Angulo AI (Hospital Central Norte Pemex, Mexico); Martínez Zarate J Jacobo, Reyes Rodriguez E, Montalvo Dominguez G (Hospital Médica Sur, Ciudad de México, Mexico); Becerra García FC (Hospital San Ángel Inn Chapultepec, Mexico); Melchor-Ruan J, Vilar-Compte D, Romero-Bañuelos E, Herrera-Gomez A, Meneses-Garcia A, Isla-Ortiz D, Salcedo-Hernández RA, Hernández-Nava JM, Morales-Castelan JE (Instituto Nacional De Cancerologia, Mexico); Sarre C, Posadas-Trujillo OE, Buerba GA, Alfaro-Goldaracena A, Pena Gomez-Portugal E, Lopez-Pena G; Hinojosa CA, Mercado MA (Universidad Nacional Autonoma de Mexico, Instituto Nacional de Ciencias Medicas y Nutricion Salvador Zubiran, Mexico City, Mexico); Ramos-De la Medina A, Martinez L, Duran I, Gonzalez DS, Martinez MJ, Nayen Sainz de la Fuente A (Hospital Español Veracruz, Mexico); Miguelena L, Hernández Miguelena L (Hospital Regional Veracruz, Mexico); Louraoui SM, El Azhari A, Rghioui M (Cheikh Khalifa International University Hospital, Morocco); Khya E (Moulay Youssef, Morocco); Ghannam A, Souadka A, El Ahmadi B, Belkhadir ZH, Majbar MA, Benkabbou A, Mohsine R (Institut National D'oncologie, Morocco); Oudrhiri MY, Bechri H, Arkha Y, El Ouahabi A (Centre Hospitalier Universitaire Ibn Sina Rabat, Morocco); Frima H, Bachiri S, Groen LC (Noordwest Ziekenhuisgroep, Alkmaar, Netherlands); Verhagen T, ter Brugge FM (Ziekenhuisgroep Twente, Almelo, Netherlands); Scheijmans JCG, Boermeester MA, Hompes R, Meima-van Praag EM, Sharabiany S (Amsterdam UMC, University of Amsterdam, Netherlands); Borgstein ABJ, Gisbertz SS, van Berge Henegouwen MI (Amsterdam UMC VUMV, Netherlands); Gans S, Van Duijvendijk P, Herklots T, De Hoop T, De Graaff MR, Sloothaak D, Bolster-van Eenennaam M, Baaij J, Klinkenbijl JHG (Gelre ziekenhuis, Apeldoorn, Netherlands); Van Eekeren R, Spillenaar Bilgen EJ (Rijnstate, Arnhem, Netherlands); Van der Burg SW, Harlaar NJ, Jonker FHW (Rode Kruis Ziekenhuis, Beverwijk, Netherlands); Vermaas M, Voigt KR (Ijsselland Ziekenhuis, Capelle aan den Ijssel, Netherlands); Nellensteijn D, Bensi EAB, (Curacao Medical Center, Willemstad, Curaçao, Curaçao, Netherlands); Posma-Bouman L (Slingeland Ziekenhuis, Netherlands); Van Sambeek M, Holscher M (Catharina Ziekenhuis, Eindhoven, Netherlands); Van den den Broek WT (St. Anna Ziekenhuis, Geldrop, Netherlands); HKruijff S, De Vries JPPM, Steinkamp PJ, Jonker PKC, Van der Plas WY, Bierman W, Janssen Y (University Medical Center Groningen, Netherlands); Franken J, Oosterling S (Spaarne Gasthuis, Netherlands); Boerma EG, Schweitzer D, Keulen MHF, Ketting S (Zuyderland MC, Sittard/Heerlen, Netherlands); Wegdam JA, Vries Reilingh TS de, Schipper E, Teeuwen PHE (Elkerliek Ziekenhuis, Helmond, Netherlands); Hendriks ER, Van Geloven AAW (Tergooi Hospital, Hilversum, Netherlands); Emous M, Poelstra R, Teunissen M (Medisch Centrum Leeuwarden, Leeuwarden, Netherlands); Gerritsen SL, Boerma D, (st. Antonius Ziekenhuis, Nieuwegein, Netherlands); De Reuver PR, Thunnissen F, Vermeulen BAM, Groen A, (Radboud Universitair Medisch Centrum, Nijmegen, Netherlands); Van Ginhoven TM, Viëtor CL, van der Oest MJW (Erasmus Medisch Centrum, Rotterdam, Netherlands); Vriens PWHE, Houwen T, J Heisterkamp (Elisabeth TweeSteden Ziekenhuis, Tilburg, Netherlands); van Petersen AS, van der Meij W, Stevens CT (Bernhoven, Uden, Netherlands); Pronk A, Bakker WJ (Diakonessenhuis, Utrecht, Netherlands); Richir MC, Vriens MR, Filipe MD, (University Medical Center Utrecht, Utrecht, Netherlands); Uittenbogaart M, Leclercq WKG, Sijmons JML, Vancoillie PJ (Maxima Medical Center, Netherlands); Konsten J, Van Heinsbergen M (VieCuri Medisch Centrum, Netherlands); Dekker NAM, den Boer FC (Zaans Medisch Centrum, Zaandam, Netherlands); Akinmade A, Adeyeye A, Enoch E, Fayose S (Afe Babalola University Multi-System Hospital, Nigeria); Okunlola AI, Adeniyi AA, Adeyemo OT, Adebara IO, Bakare A, Babalola OF, Abiyere OH, Banjo OO (Federal Teaching Hospital, Ido Ekiti, Nigeria); Olori S, Akaba OG, Agida ET, Abdullahi IH (University of Abuja Teaching Hospital, Nigeria); Egbuchulem IK, Olulana D, Lawal TA, Ogundoyin O, Oyelakin OA, Nwaorgu OG, Sule SO (University College Hospital, Nigeria); Makwe CC, Afolabi BB, Seyi-Olajide JO, Ademuyiwa AO, Bode CO, Atoyebi O, Elebute OA, Okunowo AA (Lagos University Teaching Hospital, Nigeria); Williams OM, Eke NG, Oshodi OA, Faboya OM, Adeniran AS, Omisanjo OA, Oshodi YA, Ogunyemi AA, Atobatele KM (Lagos State University Teaching Hospital, Nigeria); Adeyeye A, Aremu I, Olasehinde O, Abdur-Rahman L, Bello J, Popoola A, Sayomi TO, Raji HO, Adeleke N, Lawal B, Habeeb O, Agodirin O (University of Ilorin Teaching Hospital, Nigeria); Tolani MA, Sholadoye TT, Nwabuoku SE, Abubakar M (Ahmadu Bello University Teaching Hospital, Nigeria); Risteski T, Cvetanovska Naunova V, Jovcheski L, Lazova E (University Clinic for Pediatric Surgery, North Macedonia); Agledahl I (Hammerfest Hospital, Norway); Breuer RG (Soerlandet Hospital Kristiansand, Kristiansand, Norway); Massoud J (Khoula Hospital, Oman); Waqar SH, Rashid I, Ayubi A (Pakistan Institute of Medical Sciences, Pakistan); Bhatti ABH (Shifa International Hospital, Pakistan); Younis MU (Jinnah post graduate medical center, Karachi, Pakistan); Ghouri A, Ayub B, Sayyed RH (Patel Hospital, Pakistan); Saleem A, Turk K, Alvi A, Abassy J, Khan S, Arshad M, Ahmed K, Siddiqui T, Pirzada A (Aga Khan University, Pakistan); Kerawala AA, Jamal A (Cancer Foundation Hospital, Pakistan); Rai L, Nafees Ahmed R, Memon AS (Dr Ruth K.m. Pfau Civil Hospital, Pakistan); Qureshi AU, Ayyaz M, Umar M, Butt U, Kashif M, Khan WH, Waris Farooka M, Wasim T (Services Hospital Lahore, Pakistan); Talat N, Tahir W, Naseem J (The Children's Hospital & The Institute of Child Health Lahore, Lahore, Pakistan); Akbar A, Afroze S, Ali L, Sultan A, Ali HB (Doctors Hospital, Pakistan); Janjua MH, Janjua A, Asghar S, Farooq MS, Sarwar MZ, Naqi SA, Gondal KM (King Edward Medical University, Mayo Hospital, Lahore, Pakistan); Bukhari SI (Lady Reading Hospital, Pakistan); Tariq M (North West General Hospital and Research Centre, Pakistan); Javed S, Yaqoob E, Ashraf M, Mahmood U, Raja Shabbir K (Holy Family Hospital, Pakistan); Abukhalaf SA, Amro A (Palestine Medical Complex, Palestine); Cabada Lee JM (Complejo Hospitalario Caja Seguro Social, Panama); Aguilar A (Hospital De Especialidades Pediatricas Css, Panama); Rodriguez E (Hospital Santo Tomas, Panama); Castillo K (Hospital Irma De Lourdes Tzanetatos CSS, Panama); Cukier M, Rodriguez-Zentner H, Arrue E (Pacifica Salud Hospital, Panama); Isaacs Beron R (Hospital Regional Rafael Hernandez Css, Panama); Rodríguez Gonzalez A (Hospital de Clínicas, II Cátedra de Clínica Quirúrgica, Universidad Nacional de Asunción, Asuncion, Paraguay); Panduro-Correa V, Cornelio DK (Hospital Hermilio Valdizan Medrano, Peru); Otiniano Alvarado CE, Caballero Sarabia VD (Arzobispo Loayza National Hospital, Peru); Vasquez-Ojeda XP, Lizzetti-Mendoza G, Niquen-Jimenez M, Shu-Yip SB, León Palacios JL, Borda-Luque G (Cayetano Heredia National Hospital, Peru); Zegarra SA, Huamán Egoávil E, Suazo Carmelo C (Guillermo Almenara National Hospital, Peru); Castro de la Mata R, Rivas D, Targarona J (Clínica Delgado Auna, Peru); Trujillo Y, Olivera Villanueva M (Hospital Nacional Daniel Alcides Carrión, Peru); Lahoud-Velaochaga A, Cabillas K, Castañeda W, Colina Casas J (Hospital Nacional Sergio E. Bernales, Lima, Peru); Betalleluz Pallardel J, Camacho Zacarías F, Vélez Segura E, Cruz Condori DL (Hospital De Emergencias Jose Casimiro Ulloa, Peru); Huamán E (Hospital Guillermo Kaelin de la Fuente, Lima, Peru); Ugarte Oscco R, Vergel Cabrera C, Huamán Egoávil E (Hospital Emergencia Ate Vitarte, Lima, Peru); Carpio Colmenares YT, García Barrionuevo LA, Cárdenas Ruiz de Castilla D, Mansilla Doria P, Li Valencia MR (Clínica El Golf - Sanna, Peru); Salazar A, Sarmiento A, Díaz C, Morales E, Ore E, Zegarra H, Siccha J, Guardia M, Sandoval M, Mendiola GC, Mimbela M (Hospital Santa Rosa De Lima, Peru); Diaz-Ruiz R, Zeta LA, Cordova-Calle E, (Hospital Regional de Piura, Jose Cayetano Heredia, Peru); Nuñez HM (Hospital II-2 Tarapoto, Tarapoto, Peru); Ortiz-Argomedo MR (Hospital Belén de Trujillo, Trujillo, La Libertad, Peru); Caballero-Alvarado J, Salazar-Tantaleán A, Espinoza-Llerena R (Hospital Regional Docente de Trujillo, Trujillo, Peru); Aliaga-Ramos M (Hospital Alberto Hurtado Abadia, La Oroya - Yauli, Peru); Asodisen O, Jabagat E, Tedoy CM (Cebu City Medical Center, Cebu City, Philippines); Ramos RA, Lopez MPJ, Violago KLE, Aram R (Ospital Ng Makati, Philippines); Carlos Santos P, Filarca RFCarlos A, Santos P, Filarca RL (Medical Center Manila-ManilaMed, Manila, Philippines); Domingo EJ, Khu KJO, Lapitan MC, Sacdalan MDP, Kho MJN, Baticulon RE, Bravo SLR (Philippine General Hospital, University of The Philippines Manila, Philippines); Cueto, MAC, Ramos, CL, Fuentes, JR (José R. Reyes Memorial Medical Center, Manila, Philippines); Sadian H, Gumarao A, Barraquio A, Cruz EM, Gonzales AD (Pasig City General Hospital, Philippines); Reyes JAS, Salud JA, Tancinco EG, Rivera RD, Lim JA (The Medical City, Philippines); Barcelon JC, Chiu JA, Carballo MI (Cardinal Santos Medical Center, Philippines); Major P, Gawron I, Jach R (Jagiellonian University Medical College, Poland); Borges F, Matos Costa P, Henriques S, Rodrigues SC (Hospital Garcia de Orta, Portugal); Gonçalves N (Hospital De Braga, Portugal); Curvas JM (Hospital de Bragança, Bragança, Portugal); Cabeleira A, Branco C, Serralheiro P, Alves R, Teles T (Hospital De Cascais - Dr. José De Almeida, Portugal); Lázaro A, Canhoto C, Simões J, Costa M, Almeida AC, Nogueira O, Oliveira A, Athayde Nemésio R, Silva M, Lopes C, Amaral MJ, Valente da Costa A, Andrade R, Martins R, Guimarães A, Guerreiro P, Ruivo A, Camacho C, Duque M, Santos E, Breda D, Oliveira JM, De Oliveira Lopez AL, Garrido S, Colino M, De Barros J, Correia S, Rodrigues M (Centro Hospitalar E Universitário De Coimbra, Portugal); Cardoso P, Martins R, Teixeira J, Soares AP, Morais H, Pereira R, Revez T, Manso MI, Domingues JC, Henriques P, Ribeiro R, Ribeiro VI, Cardoso N, Sousa S, Martins dos Santos G (Centro Hospitalar Universitário do Algarve - Unidade De Faro, Portugal); Carvalho L, Osório C, Antunes J, Lourenço S, Balau P, Godinho M, Pereira A (Centro Hospitalar Entre o Douro e Vouga, Santa Maria da Feira, Portugal); Silva N, Kam da Silva Andrade A, Pereira Rodrigues A, Borges N, Correia J, Vieira I, Ribeiro T, Catarino J, Correia R, Pais F, Carreira Garcia R, Bento R, Cardoso J, Luis M, Santos E, Henriques J, Patena Forte J, Maciel J, Pinheiro Santos J, Silva M, Silva TP, Branquinho A (Centro Hospitalar Universitário Lisboa Central, Portugal); Caiado A (Instituto Português De Oncologia De Lisboa Francisco Gentil, Portugal); Miranda P, Garrido R, Peralta Ferreira M, Ascensão J, Costeira B, Cunha C, Rio Rodrigues L, Sousa Fernandes M, Azevedo P, Ribeiro J, Lourenço I, Gomes H, Mendinhos G, Nobre Pinto A (Hospital Beatriz Angelo, Portugal); Ribeiro A, Gil CG, Lima-da-Silva C, Pereira C, Tavares F, Ferraz I, Almeida JI, Marialva J, Lopes L, Costa MJMA, Nunes-Coelho M, Teixeira MJ, Machado N, Alfonso JP, Saraiva P, Silva RL, Santos R, Almeida-Reis R, Correia-de-Sá T, Fernandes V, Almeida-Pinto J, Gonçalves JP (Centro Hospitalar do Tâmega e Sousa, Portugal); Santos-Sousa H, Cavaleiro S, Leite-Moreira AM, Pereira A, Pereira-Neves A, Faria CS, Monteiro JM, Nogueiro J, Sampaio-Alves M, Magalhães Maia M, Vieira P, Pina-Vaz T, Jácome F, Devezas V, Almeida A, Silveira H, Vaz S, Castanheira Rodrigues S (Centro Hospitalar E Universitário De São João, Portugal); Costa Santos D, Grilo JV, Abreu da Silva A, Claro M, Deus AC (Hospital Do Litoral Alentejano, Portugal); Branquinho R (Centro Hospitalar Medio Tejo, Portugal); Santos PMDD, Patrício B, Vieira Paiva Lopes AC (Hospital De Torres Vedras - Centro Hospitalar Do Oeste, Portugal); Mendes JM, Carvalho MF, Oliveira CM (Centro Hospitalar do Médio Ave, Portugal); Tojal A, Pinto J (Centro Hospitalar Tondela-Viseu, Portugal); Abutaka A, Zarour A, Abdelkareem M, Ali SM, Al Tarakji M, Alfkey R, Mukhtar K, Wani IR, Singh R, Ahmed K, Bouchiba N, Mahdi H, Abdelaziem Mustafa S, Al Ansari A (Hamad General Hospital, Qatar); Drasovean R, Caziuc A (Clinica Chirurgie I, Spitalul Clinic Judetean de Urgenta, Cluj-Napoca, Romania); Galliamov E, Agapov M, Kakotkin V, Semina E, Kubyshkin V, Kamalov A (Moscow Research and Educational Center, Lomonosov Moscow State University, Russian Federation); Efetov SK, Kochetkov VS, Garmanova T, Tsarkov P, Tulina I, Rodimov S, Markaryan D, Kazachenko E (Clinic of Coloproctology and Minimally Invasive Surgery, Sechenov Medical State University, Moscow, Russian Federation); Yanishev A, Abelevich A, Bazaev A, Kokobelyan A, Zarubenko P (Privolzhsky Research Medical University, Nizhny Novgorod Regional Clinical Hospital, Russian Federation); Zakharenko A, Novikova A (Pavlov First State Medical University of St. Petersburg, Russian Federation); Kim G, Shmatov D, Stoliarov M, Kamenskikh M (Saint Petersburg State University Hospital, Saint Petersburg, Russian Federation); Nambi G (University Hospital, Saudi Arabia); Almulhim AS, Madkhali T, Alzouhir A, Alissa A (King Fahad Hospital Hofuf, Saudi Arabia); Alameer E, Badedi M, Alnami AQ, Darraj H (Jazan University - King Fahd Central Hospital - Sabia General Hospital - Baish General Hospital, Saudi Arabia); Alkhuzaie A, Khadwardi F, Abualjadayel M, Tashkandi W (King Abdulaziz Hospital and Oncology Center, Saudi Arabia); Farsi A, Malibary N, Trabulsi N, Farsi S (King Abdulaziz University Hospital, Saudi Arabia); Said bayazeed A, Nasser M, Siddiqui MS, Al Awwad S (King Fahad General Hospital, Saudi Arabia); Alshahrani M, Alsharif F, Fahmi MW (Aseer Central Hospital, Saudi Arabia); Gudal A, Alasmari A, Alqahtani S (King Abdullah Medical Complex - Jeddah, Saudi Arabia); Majrashi S, Mashat A, Al Raddadi R (East Jeddah General Hospital, Saudi Arabia); Alharbi A, Nasser Y, Hamayel H, Alhojaili A, Aljohani R (King Fahad General Hospital, Saudi Arabia); Sogair O (Ohud Hospital, Saudi Arabia); Alfarhan O, Alzahrani A, Alzomaili B, Tashkandi W (Hera General Hospital, Saudi Arabia); A Azab M (King Abdullah Medical City Makkah, Saudi Arabia); Alotaibi M, Maashi A, Zowgar A, Alsakkaf M (King Faisal Hospital, Saudi Arabia); Alnemary M, Khayat S, Felmban S, Almhmadi A (Alnoor Specialist Hospital, Saudi Arabia); Alqannas M, Cortés Guiral D, Alyami M, Elawad A (King Khalid Hospital, Najran, Saudi Arabia); Alhefdhi A, Alresaini F, Kurdi W, Tulbah M, Aldakheel M, Alsahan N, Koussayer S, Elsheikh H, Al-qattan M, Alshanafey S, Rafique A, Mahabbat N, Saeed B (King Faisal Specialist Hospital, Saudi Arabia); Al-Kharashi E, Alsowaina K, Arab N, Aljaber F, Al Hasan I, Alghamdi A, Badahdah F, Alghuliga A, Abdulfattah F, Alanazi F, Albaqami F, Alsuhaibani A (Prince Sultan Military Medical City, Saudi Arabia); AlFakhri A, Alqasem S, Alajaji N (King Fahad Medical City, Saudi Arabia); Nouh T, Bin Nasser A, Alowais J, Alburakan A, Alamri O, Albdah A, Alawi K, Alshalhoub M (King Saud University, Saudi Arabia); ElSanhoury K, Almofarreh A, Ibrahim S, Elshafie H, Osman I, Guzman T, Mutair H, Siddiqui A, Chowdhury S, Alghamdi R, Almutrafi S, Alfaifi J, D'Souza J, Alshitwi A, Alkreedees N, Alramadhan M, Alshehri M (King Saud Medical City, Saudi Arabia); Alzahrani A, Alobaysi S, Badr H, Alshahrani A (Security Forces Hospital, Saudi Arabia); Alshehri A, Alrashed M, Altahan T, Alsabahi T, Alhossaini R, Sbaih M (Prince Mohammed Bin Abdulaziz Hospital, Saudi Arabia); Alalawi Y, Alnwijy K, Al Ayed A (King Salman Armed Forces Hospital, Tabouk, Saudi Arabia); Ghedan S, Alharthi R, Awad S, I Sharara M, Abdelrhman S, Althobaiti W (King Faisal Medical Complex, Saudi Arabia); Srbinovic L, Perovic M, Mikovic Z, Nikolic B, Vasiljevic M, Pazin V, Mandic Markovic V, Dimitrijevic D, Zecevic N (Clinic for Gynecology and Obstetrics Narodni Front, Serbia); Gregoric P, Micic D, Loncar Z, Doklestic K, Ivancevic N (Clinic for Emergency Surgery, Emergency Center, Clinical Center of Serbia, Serbia); Djukic V, Stojakov D, Ilic R, Savic P, Pijanovic N, Milanovic M, Radosavljevic M, Dejanovic T, Kostic M, Paskas J, Bojic S, Stevanovic P, Djuric M (KBC Dr Dragisa Misovic-Dedinje, Serbia); Kadija M, Tulic G, Glisovic Jovanovic I (Clinic for Orthopaedic Surgery and Traumatology, Clinical Center of Serbia, Belgrade, Serbia); Lieske B (National University Hospital, Singapore, Singapore); Kayombo E, Kruger I, De Kock M, Malan A, Ferreira C, Du Preez H, Mulder W, Noel C, Le Grange S, Lusawana O (Universitas Academic Hospital Complex, South Africa); Kies C, Steyn E, Janson J, Buitendag JJP, Chu K, Mihalik M, Nel R, Naidoo S (Tygerberg Hospital, South Africa); Kloppers C, Nel D, Jonas E, Pickard H, Bernon M, Almgla N, Rayamajhi S, Mugla W (Groote Schuur Hospital, South Africa); Carapinha C (Netcare Clinton Hospital, Johannesburg, South Africa); Hyman GY, Fourtounas M, Moore R (Chris Hani Baragwanath Academic Hospital, South Africa); Sánchez Mozo A (Complejo Hospitalario Universitario De Albacete, Spain); Aguado Lopez H (Hellin Hospital, Spain); Zárate Pinedo A (Hospital Germans Trias i Pujol, Badalona, Spain); Jimenez Toscano M, Alonso de la Fuente N, Mancebo G, Cecchini L, Munarriz M, Cazador Labat M, López Campillo A, Martorell P, Espinosa CA (Hospital Del Mar, Spain); Caja Vivancos P, Villalabeitia Ateca I, Prieto Calvo M, Marín H, Martin Playa P, Gainza A, Aragon Achig EJ, Rodriguez Fraga A, Melchor Corcóstegui I, Mallabiabarrena Ormaechea G, Garcia Gutierrez JJ, Barbier L, Pesántez Peralta MA, Jiménez Jiménez M, Municio Martín JA, Gómez Suárez J, García Operé G, Pascua Gómez LA, Oñate Aguirre M (Hospital Universitario Cruces, Spain); Fernandez-Colorado A, De la Rosa-Estadella M, Gasulla-Rodriguez A, Serrano-Martin M, Peig-Font A, Junca-Marti S, Juarez-Pomes M, Garrido-Ondono S, Blasco-Torres L, Molina-Corbacho M, Maldonado-Sotoca Y, Gasset-Teixidor A, Blasco-Moreu J (Corporació Sanitària Parc Taulí, Spain); Gomez Fernandez L, Cayetano Paniagua L (Consorci Sanitari De Terrassa, Spain); Izquierdo O, Ventura D, Castellanos J (Parc Sanitari Sant Joan de Déu, Sant Boi de Llobregat, Barcelona, Spain); Ballester Vazquez E, Sanchez Lopez A, Balague Ponz C, Targarona Soler EM, Sanchez Cabús S, Molina Santos V, Gonzalez Lopez JA, Medrano Caviedes R, Moral Duarte A (Hospital De La Santa Creu I Sant Pau, Spain); Espin-Basany E, Pellino G, Blanco-Colino R (Vall D'hebron University Hospital, Spain); Turrado-Rodriguez V, Lacy AM, de Lacy FB, Morales X, Carreras-Castañer A, Torner P, Jornet-Gibert M, Balaguer-Castro M, Renau-Cerrillo M, Camacho-Carrasco P, Vives-Barquiel M, Campuzano-Bitterling B, Gracia I, Pujol-Muncunill R (Hospital Clínic de Barcelona, Spain); Martin-Sole O, Rubio-Palau J, Tarrado X, Garcia-Aparicio L, De Haro Jorge I, Martin A, Rojas-Ticona J, Perez-Bertolez S, Cuesta Argos M, Capdevila Vilaro B, Coronas Soucheiron M, Riba Martinez M, Saura Garcia L, Prat Ortells J, Bejarano Serrano M, Parri P, Massaguer C, Vicario F, Palazon Bellver P, Moraleda Gudayol I (Hospital Sant Joan de Deu de Barcelona, Spain); Lara A, Escobar D, Arrieta M, Garcia de cortazar U, Villamor Garcia I (Hospital Universitario De Basurto, Spain); Landaluce-olavarria A, Gonzalez De miguel M, Fernández Gómez Cruzado L, Begoña E, Lecumberri D (Hospital Urduliz, Spain); Acosta Mérida MA, Yepes Cano AF (Hospital Universitario De Gran Canaria Doctor Negrín, Spain); Estaire Gómez M, Padilla-Valverde D, Sánchez-García S, Sanchez-Pelaez D, Jimenez Higuera E, Picón Rodríguez R, Fernández Camuñas À, Martínez-Pinedo C, Garcia Santos EP, Muñoz-Atienza V, Moreno Pérez A, López de la Manzanara Cano CA (Hospital General Universitario De Ciudad Real, Spain); Ugarte-Sierra B, Ibáñez-Aguirre FJ, De Andres Olabarria U, Fernández Pablos FJ, Durán Ballesteros M, Sanz Larrainzar A (Hospital Universitario De Galdakao, Spain); Jiménez Carneros V, Valle Rubio A, Alonso-Lamberti L, Salazar A, García-Quijada J, Leon R, Rodriguez JL, Jimenez Miramón J, Jover JM, Martín Salamanca MB, Assaf M, Pérez Simón V, Landeo Agüero SA, Baeza Pintado N, Huertas Fernandez MA, Carabias A (Getafe University Hospital, Spain); Sosa MV, Lora-Cumplido P, Lanuza L (Hospital de Cabueñes, Spain); Galipienso Eri M, Garcia Montesino JD, Dellonder Frigolé J, Noriego Muñoz D (Hospital Universitari De Girona Dr. Josep Trueta, Spain); Navarro-Sánchez A (Complejo Hospitalario Universitario Insular-Materno Infantil de Gran Canaria, Spain); Enjuto D, Perez Gonzalez M, Díaz Peña P, Gonzalez J, Marqueta De Salas M, Martinez Pascual P, Rodríguez Gómez L, Garcés García R, Ramos Bonilla A, Herrera-Merino N, Fernández Bernabé P, Cagigal Ortega EP, Hernández I, García de Castro Rubio E, Cervera I (Severo Ochoa University Hospital, Spain); Sánchez-Guillén L, Fernández-Candela A, Curtis-Martínez C, Soler-Silva Á, Oller Á, Triguero-Cánovas D, Bosch-Ramírez M, Lillo C, Lario S, Arroyo A (Universidad Miguel Hernández, Elche, Alicante, Spain); Espino Segura-Illa M, Sánchez Aniceto G, Castaño-Leon AM, Jimenez-Roldan L, Delgado Fernandez J, Pérez Núñez A, Lagares A, Garcia Perez D, Santas M, Paredes I, Esteban Sinovas O, Moreno-Gomez L, Rubio E, Vega V, Vivas Lopez A, Labalde Martinez M, García Villar O, Pelaéz Torres PM, Garcia - Borda J, Ferrero Herrero E, Gomez P, Eiriz Fernandez C, Ojeda-Thies C, Pardo Garcia JM (12 De Octubre University Hospital, Spain); Di Martino M, De la Hoz Rodriguez A, García Septiem J, Maqueda González R, Delgado Búrdalo L, Correa Bonito A, Martin-Perez E (Hospital Universitario De La Princesa, Spain); García Villayzán JE, Albi Martin B (Fundación Jimenez Diaz University Hospital, Spain); Lozano Lominchar P, Martin L, Fernadez M, Rey-Valcarcel C, Tousidonis M, Martin-Albo Caballero L, Lowy A, Alonso Ortuño P, Ayuso Herrera E, Caño Velasco J, Aragon-Chamizo J, Perez Diaz MD, Mateo-Sierra O, Quintana-Villamandos B, Barrio JM, Fanjul M, Sanchez- Perez C, Fernandez ML, Hernandez-Kakauridze S, Rio J (Hospital General Universitario Gregorio Marañón, Spain); Díaz Pérez D, Serrano González J, Colao García L, Gutierrez Samaniego M, Hernandez Bartolome MA, Galindo Jara P, Esteban Agustí E (Hospital Universitario De Torrejón De Ardoz, Spain); Ripollés-Melchor J, Abad-Motos A, Abad-Gurumeta A, Martínez-Hurtado E, Ruiz-Escobar A (Infanta Leonor University Hospital, Spain); Brogly N, Guasch E, Hernandez Gutierrez A, Bartha Rasero JL, Perez Y, Garcia-Pineda V, Gracia M, Siegrist Ridruejo J, Diestro MD, Sanchez-Mendez JI, Marti C, Melendez M, Moreno-Palacios E, Loayza A, Frias L, Zapardiel I, Rubio-Perez I, Prieto Nieto MI, Guevara J, Gegundez Simon A, GortazarS, Chavarrias N, Alvarez E, Saavedra J, Ramos-Martin P, Urbieta A, Gomez Rivas J, Toribio-Vázquez C, Yebes A (Hospital Universitario La Paz, Spain); Hernández-García M, Losada M, Diéguez B, García-Conde M, Alonso Poza A (Hospital Universitario Del Sureste, Spain); Marquez L, Becerra R, Martin M, Jorgensen T (Hospital Central De La Cruz Roja San Jose Y Santa Adela, Spain); Muguerza JM, Dziakova J, Sánchez del Pueblo C, Saez Carlin P, Camarero E, Picazo S, Pizarro MJ, Avellana R, Catalán V, Lopez Antoñanzas L, Cano O, Anula R, Sanz Lopez R, Sanz Ortega G, Garcia Alonso M, Torres AJ, Martin Antona E, Garcia Botella S (Hospital Clínico San Carlos De Madrid, Spain); Ramos D, Barranquero AG, Ocaña J, Núñez J, Cerro Zaballos C (Hospital Universitario Ramón Y Cajal, Spain); Crego-Vita D, Huecas-Martinez M (Hospital Central De La Defensa Gomez Ulla, Madrid, Spain); Diez Alonso M, Mendoza-Moreno F, Vera Mansilla C, Ovejero Merino E, Hernandez P, Blazquez Martin A, Ruiz Grande F, Morales Palacios N, Garcia-Loarte Gomez E, Garca Rico E (Hospital Universitario Principe De Asturias, Spain); Minaya-Bravo AM, San Miguel Méndez C, Galvan Pérez A, Gonzalez-Gonzalez E, Robin Valle de Lersundi A, Calcerrada Alises E, García-Ureña MA, Cruz Cidoncha A (Hospital Del Henares. Universidad Francisco de Vitoria., Spain); Troncoso Pereira P, Alcaide Matas F, García Pérez JM (Hospital Mateu Orfila, Spain); Muñoz Vives JM, Osorio A, Gómez Díaz CJ, Guariglia CA, Soto Montesinos C, Sanchon L, Xicola Martínez M, Guàrdia N, Collera P, Diaz Del Gobbo R, Sanchez Jimenez R, Farre Font R, Flores Clotet R (Fundació Althaia - Xarxa Assistencial Universitària de Manresa, Spain); Calvo Espino P, Guillamot Ruano P (Hospital Universitario De Móstoles, Spain); Rey-Biel J, Pingarrón-Martin L, Ruiz Martin I and Moliner Sanchez C (Rey Juan Carlos University Hospital, Spain); Carrasco-Prats M, Giménez-Francés C, Ruiz-Marín M, Fernández-López AJ, García-Escudero D, García-Porcel V, Lax-Pérez R, Sánchez-Robles M, Valero-Soriano M, Medina-Manuel E, García-Soria V, Gurrea-Almela E, Marco-Garrido A, Martínez-Alonso JA, González-Valverde FM, Fernández-Fernández PV, Sánchez- Rodríguez C (Hospital General Reina Sofía, Spain); Aguilar-Jimenez J, Baeza-Murcia M, Aguayo-Albasini JL (Morales Meseguer University Hospital, Murcia, Spain); Nicolás-López T, Alconchel F (Hospital Clínico Universitario Virgen de la Arrixaca (IMIB-Arrixaca), Spain); Fernández Martínez D, Solar-Garcia L, García Flórez LJ (Hospital Universitario Central De Asturias (Huca), Spain); Llaquet Bayo H (Hospital de Palamós-SSIBE, Spain); Pujol-Cano N, Segura-Sampedro JJ, Soldevila-Verdeguer C, Jeri-McFarlane S, Gil-Catalan A, Craus-Miguel A, Cruz L, Valente P, Afonso-Garcia M, Ferrer-Inaebnit E, Oseira-Reigosa A, Fernandez-Vega L, Villalonga-Ramirez B, Gonzalez Argente FX (Son Espases University Hospital, Spain); Mora-Guzmán I (Hospital Santa Bárbara, Spain); Landete Molina FJ, Morera Ocón FJ, Canelles Corell E (Hospital General Asociado Universitario de Requena, Spain); Gavaldà Pellicé MT, Salinas Peña JR, Cavallé Busquets P (Hospital Universitari Sant Joan, Reus, Spain); Trebol J, Sánchez-Casado AB, Munoz-Bellvis L (Complejo Asistencial Universitario De Salamanca, Spain); Pérez-Sánchez LE, Concepción Martín V, Díaz García A, Vallve-Bernal M (Hospital Universitario Nuestra Señora De Candelaria, Spain); Calvo Rey A, Prada hervella GM, Dos Santos Carregal L, Rodriguez Fernandez MI, Freijeiro M, El Drubi Vega S (Hospital Clinico Universitario De Santiago De Compostela (Chus), Spain); Picardo AL, Cuadrado-Garcia A, Serralta de Colsa D, Rojo Lopez JA, Sanchez Cabezudo Noguera F, Ortega Vazquez I, Garcia-Sancho Tellez L, Mato P, Heras Aznar J (Infanta Sofia University Hospital, Spain); Jimeno Fraile J, Morales-Garcia D, Carrillo-Rivas M, Toledo Martínez E, Pascual À (Marqués De Valdecilla University Hospital, Spain); Senent-Boza A, Sánchez-Arteaga A, Benítez-Linero I, Manresa-Manresa F, Tallón-Aguilar L, Melero-Cortés L, Fernández-Marín MR, Durán-Muñoz-Cruzado VM, Ramallo-Solís I, Beltrán-Miranda P, Pareja-Ciuró F, Antón-Eguía BT (Hospital Universitario Virgen Del Rocío, Spain); Oliva Mompean F, Gomez-Rosado J, Reguera-Rosal J, Valdes-Hernandez J, Capitan-Morales L, del Toro Lopez MD (Hospital Universitario Virgen Macarena, Spain); Achalandabaso Boira M, Memba Ikuga R, Abellán M, Sales R, Olona C, Jorba R (Hospital Universitari De Tarragona Joan XXIII, Spain); Hernandez Gutierrez J, Tébar Zamora A (Complejo Hospitalario De Toledo, Spain); Sancho-Muriel J, Cholewa H, Frasson M (Hospital Universitario Y Politécnico La Fe, Spain); Domenech J, Roselló Añón A, Sangüesa MJ (Hospital Arnau De Vilanova, Spain); Moro-Valdezate D, Garcés-Albir M, Lopez F (Hospital Clínico Universitario de Valencia, Spain); Bernal-Sprekelsen JC, Catalá Bauset JC, Renovell Ferrer P, Martínez Pérez C, Gil-Albarova O, Gilabert Estellés J, Aghababyan K (Consorcio Hospital General Universitario, Spain); De Andrés-Asenjo B, Beltrán de Heredia J, Vázquez- Fernández A, Ortiz de Solorzano-Aurusa FJ, Trujillo-Díaz J, Ruiz-Soriano M, Jezieniecki C, Gómez-Sanz T, Núñez-Del Barrio H, Romero-De Diego A, García-Virto V, Aguado HJ (Hospital Clínico Universitario De Valladolid, Spain); Fernández Martín MT (Hospital Medina Del Campo, Spain); Tejero-Pintor FJ, Pérez-Saborido B, Choolani Bhojwani E, Acebes García F, Marcos-Santos P, Bueno Cañones AD, Sanchez Gonzalez J, Toledano M, Bailón M, Pacheco Sánchez D (Hospital Universitario Río Hortega, Spain); Paniagua Garcia Senorans M, Sanchez-Santos R (Álvaro Cunqueiro Hospital, Spain); Vazquez Melero A, Garcia D, Díez E, Herrero I, Soeda IM, Camuera M, Balluerca M, Sánchez-Rubio M, Paunero Vazquez P (Hospital Universitario Araba, Spain); Martinez-German A, Gracia-Roche C, Gascon-Ferrer I, Duque-Mallen V, De Miguel-Ardevines MDC, Sanchez-Fuentes N, Santero-Ramirez MS, Matute-Najarro M, Herrero-Lopez M, Sanchez-Rubio M, Cantalejo-Diaz M, Gonzalez-Nicolas-Trebol MT, Saudi-Moro S, Jariod-Ferrer UM (Hospital Universitario Miguel Servet, Spain); Rivas R, Rivas F (Hospital Clinico Universitario Zaragoza, Zaragoza, Spain); Escartin J, Blas Laina JL, Nogués A, Cros B, Talal El-Abur I, Garcia Egea J, Yanez C (Hospital Royo Villanova, Spain); Jayarajah U, Ravindrakumar S, Rodrigo VSD, Arulanantham A, Bandara GBKD (District General Hospital Chilaw, Sri Lanka); Hamid HKS, Ali EE, Widatalla ABH, Bakheit I, Awadelkarim M (Ibrahim Malik Teaching Hospital, Sudan); Ali karar AA (Al-Rajhi, Sudan); Saleh M (University of Gezira Hospital, Sudan); Taflin H (Sahlgrenska University Hospital, Sweden); Myrelid P, Amorim Braz L (Linköping University Hospital, Sweden); Hagander L, Hambraeus M, Omling E, Salö M (Lund University, Skåne University Hospital,, Sweden); Arkani S, Freedman J (Danderyds Hospital, Sweden); Montan C, Lindqvist EK, Elbe P, Hultgren R (Karolinska University Hospital, Sweden); Älgå A, Nordberg M, Sandblom G (South General Hospital, Sweden); Rutegård M, Holmner F, Sund M, Löfgren N (Umeå University Hospital, Sweden); Tampakis A, Kollmar O, von Fluee M (Clarunis, University Hospital Basel, Basel, Switzerland); Balaphas A, Toso C, Colucci N, Popeskou SG (Geneva University Hospitals, Switzerland); Gass M, Scheiwiller A, Metzger J (Luzerner Kantonsspital, Switzerland); Gialamas E, Chevallay M, Sauvain M, Dwidar O (Pourtales Neuchatel Hospital, Switzerland); Kiessling SY, Stoeckli SJ (Kantonsspital St. Gallen, Switzerland); Mongelli F, Bernasconi M, Di Giuseppe M, Christoforidis D, La Regina D, Arigoni M (Ente Ospedaliero Cantonale, Switzerland); Adamina M, Peros G, Guglielmetti L, Solimene F, Giardini M, Bächler T, Crugnale AS (Kantonsspital Winterthur, Switzerland); Gutschow CA, Turina M (Universitätsspital Zürich, Switzerland); Ersen O (Ankara University Medical School, Ankara, Turkey); Onan MA, Kozan R (Gazi University Medical Faculty Hospital, Turkey); Erol T, Dincer HA (Hacettepe University Hospital, Turkey); Yildiz A (Yildirim Beyazit University Yenimahalle Training and Research Hospital, Turkey); Iflazoglu N, Yalkın O (Bursa City Hospital, Bursa, Turkey); Isik A (Erzincan University Hospital, Turkey); Ozben V, Aytac E, Aliyeva Z, Akaydin E, Ozmen BB, Baca B (Acibadem Atakent Hospital, Turkey); Altinel Y, Calikoglu F, Tokocin M, Hacim NA, Akbas A, Meric S, Vartanoglu T, Yigitbas H, Ercetin C, Ercan G (Bagcilar Research and Training Hospital, Turkey); Ozgur I, Keskin M (Istanbul University Faculty of Medicine, Istanbul, Turkey); Saracoglu KT, Cimenoglu B, Demirhan R, Kale A, Simsek T, Gundogdu EC (Kartal Dr. Lutfi Kirdar Training and Research Hospital, Turkey); Abbasov A (Liv Hospital Ulus, Turkey); Tanal M, Citgez B, Bozkurt E, Yetkin SG, Mihmanli M (University of Health Sciences Sisli Hamidiye Etfal Training and Research Hospital, Turkey); Alhamed A, Ergun S, Sanli AN, Velidedeoglu M, OZcelik k MF, Uludag SS, Zengin AK, Cebi S, Demirkiran F, Bese T, Acikgoz AS, Kayan B, Aykanat Y, Mutlu D (Istanbul University - Cerrahpasa, Cerrahpasa Medical Faculty, Turkey); Göksoy B (Sehit Prof.Dr. İlhan Varank Training and Research Hospital, Turkey); Kara Y, Bozkurt MA, Kocatas A (Kanuni Sultan Suleyman Training and Research Hospital, Turkey); Öğücü H, Uslu G, Arican C, Tugmen C, Aydin C, Yesilyurt D, Avci EK, Kebapçı E, Kilinc G, Sert İ, Tuncer K, Akalin M, Emiroglu M, Demirli Atici S, Salimoğlu S, Kaya T, Kirmizi Y (University of Health Sciences Tepecik Training and Research Hospital, Turkey); Tatar OC, Yüksel E, Güler SA, Yildirim A, Utkan NZ, Gözal K, Köken H, Yabas A (Kocaeli University Teaching Hospital, Turkey); Gonullu E, Altintoprak F, Akin E, Kamburoglu B, Capoglu R, Kucuk F, Demir H, Cakmak G, Firat N, Celebi F, Kocer B, Mantoglu B, Bayhan Z, Dikicier E (Sakarya University Faculty of Medicine, Turkey); Colak E, Kucuk GO (Samsun Training and Research Hospital, Turkey); Karaman E, KolusabA, Karaaslan O (Van Yuzuncu Yil University, Medical Faculty, Turkey); Majid I, Alshryda S (Al Jalila Children's Speciality Hospital, United Arab Emirates); Abbas F, Abbas FMA (Dubai Hospital, United Arab Emirates); Mohammed D, Tahlak MA (Latifa Women and Children Hospital, Dubai, United Arab Emirates); Yammahi A, Albaroudi AA, Elyafawi B, Saber A, Khansaheb H, Alsaadi H, Alzarooni N (Rashid Hospital, United Arab Emirates); Bekheit M, Kamera BS, Elhusseini M, Sharma P, Ahmeidat A, Gradinariu G, Cymes W, Hannah A, Mignot G, Shaikh S, Agilinko J (Aberdeen Royal Infirmary, United Kingdom); Angelou D, Neely D, McCanny A, McAree B (Antrim Area Hospital, Northern Health and Social Care Trust, United Kingdom); Baldwin AJ, West R, Gammeri E, Catton A, Marinos Kouris S (Stoke Mandeville, Wycombe General, United Kingdom); Pereca J, Singh J (University Hospital Ayr, United Kingdom); Seymour Z, Jones R, Leeson S, Peevor R, Lala AK, Houlden C (Ysbyty Gwynedd, United Kingdom); Kahiu J, Hossain N, Hosny S (Barnet General Hospital, United Kingdom); Patel P, Handa S, Kaushal M, Kler A, Reghuram V, Tezas S (Furness General Hospital, United Kingdom); Fairhurst K, Yates C, Mitchell S, Bunni J, Richards S, George R, Lee SM, Phull J, Frost J, Burnard S, Crowley R, Airey A (Royal United Hospitals Bath, United Kingdom); Bevan K, Makin-Taylor R, Ong CS, Callan R, Bloom O (Bedford Hospital, United Kingdom); Aljanadi F, Moawad N, Jones M, Gregg A, Jeganathan R (Royal Victoria Hospital, Belfast, United Kingdom); Pachl M, Martin B (Birmingham Children's Hospital, United Kingdom); Archer JE, Odeh A, Siddaiah N (Royal Orthopaedic Hospital, United Kingdom); Singhal R, Naumann DN, Karandikar S, Syed A, Tucker ON, Alam R, Kalkat M (Heartlands Hospital, United Kingdom); Mak JKC, Kulkarni R, Sharma N, Nankivell P, Tirotta F, Parente A, Breik O, Kisiel A, Cato LD, Saeed S, Bhangu A, Griffiths E, Pathanki AM, Ford S, Desai A, Almond M, Kamal M (Queen Elizabeth Hospital Birmingham, United Kingdom); Sundar S, Leung EYL, Kaur R, Brett-Miller C, Buruiana FE (Pan-Birmingham Gynaecological Cancer Centre, United Kingdom); Markose G, De Gea Rico A, Taib A, Myatt D, Sulaiman Khaled A, Younis F (East Lancashire Hospitals NHS Trust, United Kingdom); Sultana A, Taggarsi M, Vitone L, Lambert J, Vaz OP, Sarantitis I, Shrestha D, Timbrell S, Shugaba A (Royal Blackburn Hospital, United Kingdom); Quddus B, Law J, Bittar MN, Creanga M, Elniel M, Youssef M, Ali S, Qadri ST (Blackpool Victoria Hospital, United Kingdom); Brixton G, Findlay L, Klatte T Majkowska A, Manson J, Potter R (Royal Bournemouth Hospital, United Kingdom); Oktseloglou V, Mosley F, De La Cruz Monroy MFI, Bobak P, Omar I, Ahad S, Langlands F, Brown V, Hashem M (Bradford Royal Infirmary, United Kingdom); Kennedy L, Jaunoo S, Coomber E, Williams O (Royal Sussex County Hospital, United Kingdom); Shalaby M, Rhodes HL (Bristol Royal Hospital for Children, United Kingdom); Williams A, Ridgway A, Pournaras D, Britton E, Lostis E, Ambler GK, Chu H, Hopkins J, Manara J, Chan M, Doe M, Moon RDC, Lawday S, Jichi T, Singleton W (Southmead Hospital, United Kingdom); Main B, Maccabe T, Newton C, Blencowe NS, Fudulu DP, Bhojwani D, Baquedano M, Caputo M, Rapetto F, Flannery, O, Hassan A (University Hospitals Bristol and Weston NHS Foundation Trust, United Kingdom); Coonar A, Aresu G, Smith C, Gearon D, Hogan J, Pradeep IS, Durio Yates H, Peryt A, Barrett-Brown ZM, King M, Ahmadi N, Jenkins D, Moorjani N, Taghavi F, Wells F (Royal Papworth Hospital, United Kingdom); Hardie J, Page S, Anazor F, King SD, Luck J, Kazzaz S (Frimley Park, Frimley Health NHS FT, United Kingdom); Mannion R, Stewart GD, Ramzi J, Mohan M, Singh AA, Ashcroft J, Baker OJ, Coughlin P, Davies RJ, Durst AZED, Abood A, Habeeb A, Hudson VE, Kolias A, Lamb B, Luke L, Mitrasinovic S, Murphy S, Ngu AWT, O'Neill JR, Waseem S, Wong K, Georgiades F, Hutchinson PJ, Tan XS, Pushpa-rajah J, Colquhoun A, Masterson L, Abu-Nayla I, Walker C, Balakrishnan A, Rooney S, Irune E, Byrne MHV, Durrani A (Addenbrooke's Hospital, Cambridge University Hospitals NHS Foundation Trust, United Kingdom); Simoes A, Eddy B, Streeter E, Ahmed I, Yao M, Wang W, Djouani A, Tait-Bailey J, Thomas M, Hassan F, Kommu S (Kent and Canterbury Hospital, United Kingdom); Chopra S (University Hospital Llandough, United Kingdom); Richards T, Sethuraman Venkatesan A, Combellack T, Williams J, Tahhan G, Mohammed M, Kornaszewska M, Valtzoglou V, Deglurkar I, Rahman M, Von Oppell U, Mehta D, Koutentakis M, Syed Nong Chek SAH, Hill G, Morris C, Shinkwin M, Torkington J, Cornish J (University Hospital of Wales, United Kingdom); Houston R, Mannan S, Ayeni F, Tustin H, Bordenave M, Robson A (Cumberland Infirmary, United Kingdom); Dovell G, Preece R, Rolland P (Cheltenham General Hospital, United Kingdom); Miranda BH (St Andrew’s Centre for Plastic Surgery and Burns, Chelmsford, United Kingdom); Sobti A, Khaleel A, Unnithan A, Memon K, Pala Bhaskar RR, Maqboul F, Kamel F, Al-Samaraee A, Madani R, Kumar L, Nisar P, Agrawal S (Ashford and St Peter's Hospital, United Kingdom); Vimalachandran D, Manu N, Eardley N, Krishnan E, Serevina OL, Martin E, Smith C, Jones A, Roy Mahapatra S, Clifford R (Countess of Chester Hospital, United Kingdom); Jones GP, Gardner A, Tripathi SS, Greenhalgh MS (Lancashire Teaching Hospitals NHS Foundation Trust, United Kingdom); Matthews W, Mohankumar K, Khawaja I, Palepa A, Doulias T (Colchester University Hospital, United Kingdom); Gill C, Dunne N, Sarma DR, Godbole C, Carlos W, Tewari N, Jeevan D, Naredla P, Khajuria A, Connolly H, Robertson S, Sweeney C, Di Taranto G, Shanbhag S, Dickson K, McEvoy K, Skillman J, Sait M, Al-omishy H, Baig M, Heer B (University Hospitals Coventry and Warwickshire NHS Trusts, United Kingdom); Brown A (Darlington Memorial Hospital, United Kingdom); Ebrahim A, Alwadiya A, Goyal A, Phillips A, Bhalla A, Demetriou C, Grimley E, Theophilidou E, Ogden E, Malcolm FL, Davies-Jones G, Ng JCK, Mirza M, Hassan M, Elmaleh N, Daliya P, Williams S, Bateman A, Chia Z (Royal Derby Hospital, United Kingdom); Premakumar Y, Jauhari Y, Koshnow Z, Bowen D, Uberai A, Hirri F, Stubbs BM (Dorset County Hospital, United Kingdom); Crichton R, Sonksen J, Aldridge K (Russell's Hall Hospital, Dudley, United Kingdom); McDonald C, Manickavasagam J, Ragupathy K, Davison S, Dalgleish S, McGrath N, Kanitkar R, Payne CJ, Ramsay L (Ninewells Hospital, United Kingdom); Ng CE, Collier T, Khan K, Evans R (University Hospital North Durham, United Kingdom); Brennan C, Henshall DE, Drake T, Harrison EM, Zamvar V, Tambyraja A, Skipworth RJE, Linder G, McGregor R, Brennan P, Mayes J, Ross L, Smith S, White T, Jamjoom AAB, Pasricha R (Royal Infirmary of Edinburgh, United Kingdom); Gallagher K, Swan R, Paterson H, Maeda Y, Kwok AMF, Tsiaousidou A, Vaughan-Shaw PG, Boyle C, Fernando D, Tham D, Leung S, Laird A (Western General Hospital, United Kingdom); Holme T, Abbott S, Razik A, Thrumurthy S, Steinke J, Baker M, Howden D, Baxter Z, Osagie L, Bence M (Epsom & St Helier University Hospitals NHS Trust, United Kingdom); Fowler GE, Massey L, Rajaretnam N, Evans J, John J, Goubran A, Campain N, McDermott FD, McGrath JS, Ng M, Pascoe J, Phillips JRA, Daniels IR (Royal Devon and Exeter Hospital, United Kingdom); A'Court J, Konarski A, Faulkner G (Royal Bolton Hospital, United Kingdom); Emerson H, Vejsbjerg K, Pearce L, McCormick W, Fisher A, Singisetti K, Aawsaj Y, Barry C (Gateshead Health NHS Foundation Trust, United Kingdom); Bajomo O, Rizvi S, Grimes C, Dusu K, Tint PY (Medway Hospital, United Kingdom); Kirk A, Irvine V (Golden Jubilee National Hospital, United Kingdom); Lammy S, O'Kane R (Royal Hospital for Sick Children, United Kingdom); Elliott L, Mccabe G, Holroyd D, Jamieson NB (Glasgow Royal Infirmary, United Kingdom); Geddes A, McMahon J, McCaul J, Al-Azzawi M, Aitken E, Glen P, Sinan LOH, Lammy S, Grivas A, Tilling EJ (Queen Elizabeth University Hospital, United Kingdom); Brown O, Boal M, Dean H, Higgs S, Stanger S, Abdalaziz H, Constable J, Ishii H, Preece R, Dovell G, Gopi reddy R (Gloucestershire Royal Hospital, United Kingdom); Madhuri TK, Tailor A, Flavin M, Walker D, Humphries S, Assalaarachchi H (Royal Surrey NHS Foundation Trust, United Kingdom); Curl-Roper T, Westwood E, Delimpalta C, Liao CCL, Velchuru V (James Paget University NHS Foundation Trust Hospital, United Kingdom); Raptis DA, Pollok JM, Machairas N, Davidson B, Fusai G, Soggiu F, Xyda S, Hidalgo Salinas C, Tzerbinis H, Pissanou T, Gilliland J, Chowdhury S, Varcada M, Hart C, Mirnezami R, Knowles J, Angamuthu N (Royal Free Hospital, United Kingdom); Vijay V, Shakir T, Hasan R, Tansey R (Princess Alexandra Hospital, Harlow, United Kingdom); Hardie C, Powell-Smith E (Harrogate District Hospital, United Kingdom); Kashora F, Siddique MH, Singh A, Barmpagianni C, Basgaran A, Basha A, Okechukwu V, Bartsch A, Gallagher P, Maqsood A, Sahnan K, Leo CA, Lewis SE, Ubhi HK, Exley R, Khan U, Shah P, Saxena S, Zafar N, Abdul-Jabar H (Northwick Park Hospital, United Kingdom); Patel M, Shabana A, Alanbuki A, Usman O (Brighton and Sussex NHS Trust, Princess Royal Hospital, United Kingdom); Ong CT, Butterworth W, Budha Magar O, El Hadi M, Abas S, Annett J (Hereford County Hospital, United Kingdom); Ross E, Loubani M, Wilkins A, Cao H, Capitelli-McMahon H, Hitchman L, Ikram H, Andronic A, Aboelkassem Ibrahim A, Totty J (Hull University Teaching Hospitals NHS Trust, United Kingdom); Blanco J, Vanker R, Ghobrial M, Jones G, Kanthasamy S, Fawi H, Awadallah M, Chen F, Cheung J (Hinchingbrooke Hospital, United Kingdom); Moscalu A, Bhuvanakrishna T, Bibby L, Sinclair M (Ipswich Hospital, United Kingdom); Nahid MAK, Williams L, Basnyat PS, Shrestha AK (William Harvey Hospital, United Kingdom); Kumaran NK, Sambhwani S, Sheikh NA, Taylor OM (Kettering General Hospital, United Kingdom); Liew I, Al-Sukaini A, Mediratta S, Saxena D (Queen Elizabeth Hospital King's Lynn, United Kingdom); Sgrò A, Rashid MM, Milne K, McIntyre J, Akhtar MA, Turnbull A, Brunt A, Stewart KE (Victoria Hospital Kirkcaldy, United Kingdom); Wilson MSJ, Rutherford D, McGivern K, Massie E (Forth Valley Royal Hospital, United Kingdom); Ho M, Wade RG, Johnstone J, Bourke G, Brunelli A, Elkadi H, Otify M, Pompili C, Burke JR, Bagouri E, Chowdhury M, Abual-Rub Z, Kaufmann A, Munot S, Lo T, Young A, Kowal M, Wall J, Peckham-Cooper A (The Leeds Teaching Hospitals NHS Trust, United Kingdom); Layton GR, Karki B, Jeong H, Pankhania S, Asher S, Folorunso A, Mistry S, Singh B, Winyard J, Mangwani J (Leicester General Hospital, United Kingdom); Caruana EJ, Mohammad A, Acharya M, Chandarana K, Ang K, Chowdhry MF, Rathinam S, Nakas A (Glenfield Hospital, United Kingdom); Boddy A, Hossain T, Ashmore C, Annamalai S, Kourdouli A, Irvine E, Al-Harbawi A, Kassam K, Al-Harbawee A, Miller A, Mair M (Leicester Royal Infirmary, United Kingdom); Lunevicius R, Sheel ARG, Sundhu M, Santini AJA, Fathelbab MSAT, Hussein KMA, Nunes QM, Jones RP, Shahzad K, Haq I, Baig MMAS, Hughes JL, Kattakayam A, Rajput K, Misra N, Shah SB, Clynch AL, Georgopoulou N, Sharples HM, Apampa AA, Nzenwa IC, Sud A (Aintree Hospital, Liverpool University Hospitals NHS Foundation Trust, United Kingdom); Harky A, Kirmani BH, Shackcloth M (Liverpool Heart and Chest Hospital, United Kingdom); Jenkinson MD, Zakaria R, Elmoslemany T, Millward CP (The Walton Centre NHS Foundation Trust, United Kingdom); Baron R, Dunne D, Szatmary P, Thomas A, McNicol F, Gahunia S, Sochorova D, Nita GE, McKinney R, Russ J, Tan JR (Royal Liverpool University Hospital, United Kingdom); Harwood R, Corbett H J (Alder Hey In The Park, United Kingdom); Rossborough C, Skelly BL (Altnagelvin Area Hospital, United Kingdom); Che Bakri NA, Nazarian S, Vashisht R, Jiao L, Jawad Z (BUPA Cromwell Hospital, United Kingdom); Allan AY, Kontovounisios C, Grove T, Warren O, Fadel MG (Chelsea and Westminster Hospital, United Kingdom); Chatzikonstantinou M, Sorelli P, Rahman S, Hadjipavlou M (Queen Elizabeth Hospital, Woolwich, United Kingdom); Holbrook C, Chong C, Kufeji D (Evelina London Children's Hospital, London, United Kingdom); Rufai SR, Lloyd IC, James G, Chari A, Silva AHD (Great Ormond Street Hospital for Children, United Kingdom); Stroman L, Challacombe B, Sayasneh A, Najdy M, Billè A, Fraser S, Agoston P, Rizzo V, King J, Nath R, McCrindle Sarah, Mehra G, Harrison-Phipps K, Pilling J, Okiror L, Routledge T, Mills L, Wali A, El-Boghdadly K (Guy's Hospital, United Kingdom); Fotopoulou C, Saso S, Fehervari M, Ploski J, Ghaem-Maghami S, Spalding D, Rajagopal P, Pai M, Habib N, Jawad Z, Hamrang-Yousefi S, Jiao L (Hammersmith Hospital, United Kingdom); Tayeh S, Chase T, Humphreys L, Ayorinde J, Ghanbari A, Cuming T (Homerton University Hospital, United Kingdom); Anscomb N, Baldwin-Smith R, Rizk M, Grainger C, Davies M, A Surendran, Nunoo-Mensah JW (King's College Hospital, United Kingdom); Dunstan M, Beak P, Gerogiannis I (Kingston Ηospital NHS Foundation Trust, United Kingdom); Jain A, Menon A, Pramodana B, Choi D, Marcus HJ, Webber L, May R, Hutchison R, Luoma V (The National Hospital for Neurology and Neurosurgery, United Kingdom); Ranjit S, Parakh J, Sarodaya V, Daadipour A, Khalifa M (Newham University Hospital, United Kingdom); Bosch KD, Bashkirova V, Dvorkin LS, Kalidindi VK (North Middlesex University Hospital, London, United Kingdom); Dudek J, Singhal T, El-Hasani S (Princess Royal University Hospital, United Kingdom); De Souza A, Cannoletta M, Rochon M, Bhudia S (Royal Bromtpon Hospital, London, United Kingdom); Bennett S, Navaratne L, Venn M, Yip V, Kayani B, Sohrabi C, Kocher HM, Minicozzi A, Banerjee A, Sullivan T, Sivaprakasam R, Anzak A, Ghufoor K, Thaha MA, Knowles C, Ledesma FS, Patki P, Popova D, Sadigh P, Ramamoorthy R, Uff C, Attwell L, Tanabalan C, Goh MA, Jayasinghe JD, Leal Silva I, Thakur B, Lebe M, Thet MS, Hughes F, Rahman R, Fuwa O (Royal London Hospital, United Kingdom); Sanders J, Oo A, Bueser T, Curtis M, Stamenkovic SA (Cardiac Unit, St Bartholomew's Hospital, United Kingdom); Abbott T, Anwar S, Sohrabi C (St Bartholomew's Hospital, United Kingdom); Williams K, Chung E, Hagger R, Karim A, Hainsworth A, Flatman M, Trompeter A, Hing C, Brown O, Tsinaslanidis P, Benjamin MW, Leyte A, Tan C, Smelt J, Vaughan P, Santhirakumaran G, Hunt I, Raza M, A Labib (St George's Hospital, United Kingdom); Luo X, Sudarsanam A, Rolls A, Lyons O, Onida S, Shalhoub J, Sugand K, Park C, Sarraf KM, Erridge S, Kinross J, Denning M, Yalamanchili S, Abuown A, Ibrahim M, Martin G (St Mary's Hospital, United Kingdom); Davenport D, Wheatstone S (St Thomas' Hospital, United Kingdom); Kasivisvanathan V, Kapriniotis K, Elhamshary A, Imam SMB (University College London Hospital At Westmoreland Street, United Kingdom); Kalavrezos N, Sinha D, Chand M Green L, Beech N, McEwen R, Kiconco H (University College London Hospital, United Kingdom); Andreani SM, Bath MF, Sahni A, Judkins N, Rigueros Springford L, Sohrabi C, Bacarese-Hamilton J, Taylor FG, Patki P, Tanabalan C (Whipps Cross University Hospital, United Kingdom); Parmar C, Mccluney S, Shah S (The Whittington Hospital, United Kingdom); Talwar R, Patel K, Askari A, Jambulingam P S, Shaw S, Maity A, Hatzantonis C, Sagar J, Kudchadkar S, Cirocchi N, Chan CH (Luton and Dunstable University Hospital, United Kingdom); Reynolds J, Alexander ME, Smart CJ (Macclesfield District General Hospital, United Kingdom); Jayasankar B, Balasubramaniam D, Abdelsaid K, Mundkur N, Gallagher B (Tunbridge Wells Hospital, United Kingdom); Shah J, Anthoney J, Emmerson O (North Manchester General Hospital, United Kingdom); Stylianides N, Abdalla M, Newton K, Bhatia K, Edmondson R, Abdeh L, Jones D, Zeiton M, Ismail O, Naseem H, Advani R (Manchester Royal Infirmary, United Kingdom); Duff S, Moura F, Brown BC, Khan A, Asaad P, Wadham B, Aneke IA, Collis J, Warburton H (Wythenshawe Hospital, Manchester University NHS Foundation Trust, United Kingdom); Fell A, Smith A, Halkias C, Evans J, Nikolaou S, English C, Kristinsson S, Oni T, Ilahi N, Ballantyne K, Woodward Z, Merh R (Queen Elizabeth The Queen Mother Hospital Margate, United Kingdom); Dunning J, Viswanath Y, Freystaetter K, Dixon J, Hadfield JN, Hilley A, Egglestone A, Smith B (James Cook University Hospital, United Kingdom); Hine T, Keeler B, Soulsby RE, Taylor A (Milton Keynes University Hospital, United Kingdom); Davies E, Ryska O, Raymond T, Rogers S, Tong A, Hawkin P (Royal Lancaster Infirmary, United Kingdom); Tingle S, Abbadessa F, Sachdeva A, Rai B, Chan CD, McPherson I, Booth K, Mahmoud Ali F, Pandanaboyana S, Grainger T, Nandhra S, Patience A, Rogers A, Roy C, Williams T, Dawe N, McCaffer C, Riches J, Bhattacharya S, Moir J, Kalson NS, Elamin Ahmed H, Mellor C, Saleh C, Koshy RM, Hammond J, Sanderson L, Wahed S, Phillips AW, Ghosh K (Newcastle Upon Tyne Hospitals NHS Foundation Trust, United Kingdom); Tang A, Beamish AJ, Price C, Bosanquet D, Magowan D, Solari F, Williams G, Nassa H, Smith L (Royal Gwent Hospital, United Kingdom); Robertson-Smith B, Mahmoud A, Ameerally P, Finch JG, Gnanachandran C, Pop I, Rogers M, Yousef Y, Mohamed I, Woods R, Zahid H, Mundy G (Northampton General Hospital, United Kingdom); Youssef M, Sreedharan L, Baskaran D, Shaikh I, Seebah K, Reid J, Watts D, Kouritas V, Chrastek D, Maryan G, Gill DF, Khatun F (Norfolk and Norwich University Hospital, United Kingdom); Gajjar K, Williamson K, Bratt D, Konstantinidi K, Walton T, Burnside N, Weaver H, Hawari M, Addae-Boateng E, Rollett RA, Collins ML, Tamimy MS, Riyat H, Wen J, Neil-Dwyer J (Nottingham City Hospital, United Kingdom); Brewer H, Humes D, Worku D, Chowdhury A, Oyende O, Lewis-Lloyd C, Adiamah A, Koh A, Jackman J, Vohra R, Navarro A, Reilly J (Queens Medical Centre, United Kingdom); Aujayeb A, Townshend D, McLarty N, Shenfine A, Jackson K, Johnson C (Northumbria NHS Hospital Trust, United Kingdom); Dass D, Ford D (Robert Jones and Agnes Hunt Orthopaedic Hospital, United Kingdom); Winter SC, Belcher E, Stavroulias D, Di Chiara F, Wallwork K, Qureishi A, Lami M, Sravanam S, Mastoridis S, Shah K, Chidambaram S, Smillie R, Shaw AV, Bandyopadhyay S, Cernei C, Bretherton C, Jeyaretna D, Ganau M, Piper RJ, Duck E, Brown S, Jelley C, Tucker SC, Bond-Smith G, Griffin XL, Tebala GD, Neal N, Vatish M, Noton TM, Ghattaura H, Maher M, Fu H, Risk OBF, Soleymani majd H, Sinha S, Shankar S, Aggarwal A, Khatkar H, Lakhoo K, Verberne C, Mastoridis S (Oxford University NHS Foundation Trust, United Kingdom); Dean B, Luney C, Myatt R, Williams MA, McVeigh J (Nuffield Orthopaedic Centre, United Kingdom); Rogers LJ, Labib PL, Miller D, Minto G, Hope N, Marchbank A, Emslie K, Panahi P, Ho B, Perkins C, Clough E, Roy H, Enemosah I, Campbell R, Natale J, Gohil K, Rela M, Raza N, (Derriford Hospital, United Kingdom); Biliatis I (Poole Hospital, United Kingdom); Khan J, Thiruchandran G, Toh SKC, Ahmad Y, Allana A, Bellis C, Babawale O, Phan YC, Lokman U, Ismail M, Koc T, Witek A, Duggleby L, Shamoon S, Stefan S, Clancy H (Queen Alexandra Hospital, United Kingdom); Chadha R, Middleton SB, Wilmott K, Hayden C, Mclaren C, Sutton J, Whyte A (Royal Berkshire Hospital, United Kingdom); Belgaumkar A, Day A, Gilbert C, Oyewole B, Narayan P, Dent H, Sandhya A, De Silva T, Waheed S (Surrey and Sussex Healthcare NHS Trust, United Kingdom); Day A, Kapoor K, Belgaumkar AP, Narayan P, Fahim M, (East Surrey Hospital, United Kingdom); Gala T, Mithany R, Morgan R, Abdelkarim M, Ibrahim S, Maw A, Asqalan A, Sundaram Venkatesan G (Glan Clwyd Hospital, United Kingdom); Singh S, Mukherjee S, Ferguson D, Smith C, Mansuri A, Thakrar A, Wickramarachchi L, Cuthbert R, Sivayoganathan S, Chui K, Karam E, Dott C, Shankar S (Queen's Hospital Romford, United Kingdom); Madhvani K, Hampton M, Hormis AP (Rotherham District General Hospital, United Kingdom); Thomas M, Pearce L, Fountain DM, Laurente R, Sigamoney KV, Dasa M, George K, Naqui Z, Galhoum M, Lipede C, Gabr A, Radhakrishnan A, Hasan MT, Kalenderov R, Pathmanaban O, Colombo F, Chelva R (Salford Royal NHS Foundation Trust, United Kingdom); Branagan G, Longstaff L, Ding D, Barlow C, Foster J (Salisbury NHS Foundation Trust, United Kingdom); Edwards J, Ward A, Tadross D, Majkowski L, Blundell C, Forlani S, Nair R, Guha S, Brown SR, Steele C, Kelty CJ, Newman T, Lee M, Chetty G, Lye G, Balasubramanian SP, Sureshkumar Shah N, Sherif M, Al-mukhtar A, Whitehall E, Giblin A, Wells F, Sharkey A, Adamec A, Madan S (Sheffield Teaching Hospital NHS Foundation Trust, United Kingdom); Narice B, Sterrenburg M, Thompson A, Varley I, Stavrakas M, Rominiyi O, Ray J, Adamec A, Crank M, Bacon A, Al-Tamimi Y, Catto J, Saad S, Abd Kahar NN, Sinha S (Royal Hallamshire Hospital, United Kingdom); Sou A, Simpson D, Hamilton E, Blair J (Shrewsbury and Telford Hospitals, United Kingdom); Jallad S, Lord J, Anderson C, El Kafsi J, Logishetty K, Saadya A, Midha R, Ip M, Subbiah Ponniah H, Stockdale T, Bacarese-Hamilton T, Foster L, James A, Anjarwalla N, Marujo Henriques D, Hettige R, Baban C, Tenovici A, Salerno G (Wexham Park, Frimley Health NHS FT, United Kingdom); Singh R, Lane J, Colvin HV, Badran A, Cadersa A, Williams S, Cumpstey A, Hamady Z, Aftab R, Wensley F, Byrne J, Morrison-Jones V, Sekhon GK, Shields H, Shakoor Z, Yener A, Talbot T, Khan A, Alzetani A, Cresner R (Southampton General Hospital, United Kingdom); Babu BHB, Liyanage ASD, Newman S, Blake I, Weerasinghe C (Southport and Formby District General Hospital, United Kingdom); Baumber R, Parry J (Royal National Orthopaedic Hospital, United Kingdom); Menakaya C, Webb JI, Antar M, Modi N, Sofat R, Noel J, Nunn R, Adegbola S, Eriberto F, Sharma V, Tanna R, Lodhia S (East and North Hertfordshire NHS Trust, Lister Hospital, United Kingdom); Johnson D, Hughes I, Hall J, Rooney J, Chatterji S, Zhang Y, Owen R, Rudic M, Hunt J (Stepping Hill Hospital, United Kingdom); Zakai D, Thomas M, Aladeojebi A, Ali M, Gaunt A, Barmayehvar B, Kitchen M, Gowda M, Mansour F, Jarvis M, Halliday E, Lefroy R, Nanjaiah P, Ali S, Kitchen M (Royal Stoke University Hospital, United Kingdom); Lin DJ, Rajgor AD, Scurrah RJ, Kang C, Watson LJ, Harris G, Royle T, Cunningham Y, James G, Steel B, Luk ACO (Sunderland Royal Hospital, United Kingdom); Boulton AJ, Khan T, Bakolas G, Ahmed I (Good Hope Hospital, United Kingdom); Herrod P, Gemmill E, Boyd-Carson H, Jibreel M, Lenzi E, Saafan T, Sapre D (King's Mill Hospital, United Kingdom); Li Z, Parkins K, Spencer N, Harries R, Egan RJ, Motter D, Jenvey C, Mahoney R, Fine N, Minto T, Henry A (Morriston Hospital Swansea, United Kingdom); Hollyman M, Grieco C, Gemmell C (Musgrove Park Hospital, United Kingdom); Whitmore H, Babar MS, Goodrum S, Scott R, Collard B, Lau K, Thomas E, Patel A, Allison J, Bowen J (Torbay and South Devon NHS Trust, United Kingdom); Dias A, Mahendran B, Gopalswamy S (Royal Cornwall Hospital, Truro, United Kingdom); Patil S, Scott L, Sarveswaran J, Michel M, Ravindran S (Pinderfields Hospital, United Kingdom); Subba K, Abou-Foul AK, Khalefa M, Hossain F, Moores T, Pickering L (Walsall Manor Hospital NHS Trust, United Kingdom); Stables G, Doorgakant A, Thiruvasagam VG, Carter J, Reid S, Mohammed R, Marlow W (Warrington & Halton Teaching Hospitals NHS Trust, United Kingdom); Ferguson H, Wilkin R, Konstantinou C, Yershov D, Vatish J, Denning A (South Warwickshire NHS Foundation Trust, United Kingdom); Shah HB, Cross GWV, Seyed-Safi P, Smart YW, Kuc A, Al-Yaseen M (Watford General Hospital, United Kingdom); Olivier J, Hanna M, Eskander P, Duncan R, Halaseh S (Weston General Hospital, United Kingdom); Das R (Hampshire Hospitals NHS Trust, United Kingdom); Wynn Jones H, Divecha H, Whelton C, Board T (Wrightington, Wigan & Leigh NHS Foundation Trust, United Kingdom); Powell S, Magee C, Agarwal K, Mangos E, Nambirajan T (Wirral University Teaching Hospital, United Kingdom); Vidya R, Chauhan G, Kaur J, Burahee A, Bleibleh S, Pigadas N, Snee D, Bhasin S, Crichton A, Habeebullah A, Bodla AS, Yassin N, Mondragon M, Dewan V (Royal Wolverhampton NHS Trust, United Kingdom); Flindall I, Mahendran V, Hanson A (Worcestershire Royal Hospital, United Kingdom); Jenner E, Richards J, Thomas-Fernandez K, Wall R (Worcestershire Acute Hospitals NHS Trust, United Kingdom); Alqallaf A, Ben-Sassi A, Mohamed I, Mellor K, Joshi P, Joshi Y (Wrexham Maelor Hospital, United Kingdom); Young R, Miu V, Sheridan K, MacDonald L, Green S, Onos L (York Teaching Hospitals NHS Trust, United Kingdom); Wong JJ, Napolitano L, Hemmila M (Michigan Medicine, United States); Amin D, Abramowicz S, Roser SM (Emory University, United States); Olson KA, Riley C, Heron C, Cardenas T, Leede E, Thornhill M, Haynes AB, McElhinney K, Roward S, Trust MD, Hill CE, Teixeira PG (Dell Seton Medical Center at the University of Texas at Austin, United States); Etchill E, Stevens K, Ladd MR, Long C, Rose J, Kent A, Yesantharao P, Vervoort D, Jenny H, Gabre-Kidan A, Margalit A, Tsai L, Malapati H, Yesantharao L (Johns Hopkins Hospital, United States); Abdou H, Diaz J, Richmond M, Clark J, O'Meara L, Hanna N (University of Maryland Medical Center, United States); Ying Y, Fleming J, Ovaitt A, Gigliotti J, Fuson A (University of Alabama Birmingham, United States); Cooper Z, Salim A, Hirji SA, Brown A, Chung C, Hansen L, Okafor BU, Roxo V, Raut CP, Jolissaint JS, Mahvi DA (Brigham and Women's Hospital, United States); Kaafarani H, Breen K, Bankhead-Kendall B, Alser O, Mashbari H, Velmahos G, Maurer LR, El Moheb M, Gaitanidis A, Naar L, Christensen MA, Kapoen C, Langeveld K, El Hechi M, Mokhtari A (Massachusetts General Hospital, United States); Haqqani MH, Drake FT (Boston Medical Center, United States); Goldenberg-Sandau A, Galbreath B (Cooper University Hospital, United States); Reinke C, Ross S, Thompson K, Manning D, Perkins, R (Atrium Health Carolinas Medical Center, United States); Eriksson E, Evans H (Medical University of South Carolina, United States); Masrur M, Giulianotti P, Benedetti E (University of Illinois at Chicago, Chicago, IL, United States); Chang G, Ourieff J, Dehart D (Mount Sinai Hospital, United States); Dorafshar A, Price T, Bhama AR, Torquati A, Cherullo E, Kennedy R, Myers J (Rush University Medical Centre, United States); Rubin K (University Hospitals of Cleveland, United States); Ban VS, Aoun SG, Batjer HH, Caruso J (University of Texas Southwestern, United States); Carmichael H, Velopulos CG, Wright FL, Urban S, McIntyre Jr RC, Schroeppel TJ, Hennessy EA, Dunn J, Zier L (University of Colorado Hospital/Memorial Hospital/Medical Center of the Rockies, United States); Burlew C, Coleman J (Denver Health, United States); Colling KP (Saint Mary's Medical Center-Essentia Health, United States); Hall B, Rice HE, Hwang ES, Olson SA, Moris D (Duke University Medical Center, United States); Verma R, Hassan R (Nassau University Medical Center, United States); Volpe A, Merola, S (NewYork Presbyterian Queens, Flushing, United States); O'Banion LA, Lilienstein J, Dirks R (University of California San Francisco-Fresno, United States); Marwan H, Almasri M, Kulkarni G, Mehdi M, Abouassi A, Abdallah M, San Andrés M, Eid J, Aigbivbalu E, Sundaresan J, George B (University of Texas Medical Branch, United States); Ssentongo A, Ssentongo P, Oh JS, Hazelton J, Maines J, Gusani N, Garner M, Horvath S (Pennsylvania State University, United States); Zheng F (Houston Methodist Hospital, United States); Ujiki M (Northshore University Healthsystem, United States); Kinnaman G, Meagher A, Sharma I, Holler E (Iu Health Methodist Hospital, United States); McKenzie K, Chan J, Fretwell K, Nugent III W, Khalil A, Chen D, Post N, Rostkowski T, Brahmbhatt D (Jamaica Hospital, United States); Huynh K, Hibbard ML (Kaiser Permanente West Los Angeles, United States); Schellenberg M (Lac+Usc Medical Center, United States); Martin RCG, Bhutiani N (University of Louisville Hospital and Norton Hospital, Louisville, United States); Giorgakis E, Laryea J, Bhavaraju A, Sexton K, Roberts M, Kost M, Kimbrough M, Burdine L, Kalkwarf K, Robertson R (University of Arkansas for Medical Sciences, United States); Gosain A, Camp L, Lewit R (Le Bonheur Children's Hospital, United States); Kronenfeld JP, Urrechaga E, Goel N, Rattan R, Hart V, Allen M, Gilna G (Jackson Memorial Hospital, United States); Cioci A, Ruiz G, Allen M, Rakoczy K, Pavlis W, Saberi R (University of Miami Hospital, United States); Morris R, Karam BS (Froedtert Hospital, United States); Brathwaite CEM, Liu H, Petrone P, Hakmi H, Sohail AH, Baltazar G, Heckburn R (NYU Langone Health-NYU Winthrop Hospital, United States); Nygaard RM, Colonna ET, Endorf FW, Hill MJ (Hennepin Healthcare, Minneapolis, United States); Maiga A, Dennis B, Levin JH, Lallemand M (Vanderbilt University Medical Center, United States); Choron R, Peck G, Soliman F, Rehman S (Robert Wood Johnson University Hospital, United States); Glass N, Juthani B, Deisher D (The University Hospital, United States); Ruzgar NM, Ullrich SJ, Sion M (Yale New Haven Hospital, United States); Paranjape C, El Moheb M, Kar AR (Newton Wellesley Hospital, United States); Gillezeau C, Rapp J, Taioli E, Miles BA, Alpert N (Mount Sinai Hospital, United States); Podolsky D, Coleman NL, Callahan MP (NewYork-Presbyterian / Columbia University Medical Center, United States); Ganly I, Brown L (Memorial Sloan Kettering Cancer Center, United States); Monson JRT (AdventHealth Orlando, Orlando, United States); Dehal A (Kaiser Permanente Panorama City Medical Center, United States); Abbas A, Soliman A, Kim B, Jones C, Dauer, MD E, Renza-Stingone E, Hernandez E, Gokcen E, Kropf E, Sufrin H, Hirsch H, Ross H, Engel J, Sewards J, Diaz J, Poggio J, Sanserino K, Rae L, Philp M, Metro M, McNelis P, Petrov R, Rehman S, Pazionis T (Temple University Hospital, United States); Till B, Lamm R, Rios-Diaz AJ, Palazzo F (Thomas Jefferson University Hospital, United States); Rosengart M, Nicholson K (University of Pittsburgh Medical Center, United States); Carrick MM, Rodkey K (Medical City Plano, United States); Suri A, Callcut R (UC Davis Medical Center, United States); Nicholson S, Talathoti N (UT Health San Antonio (UTHSA), University Hospital (UHS), San Antonio, United States); Klaristenfeld D (Kaiser Permanente San Diego Medical Center, United States); Biffl W, Marsh C, Schaffer K (Scripps Memorial Hospital, United States); Berndtson AE, Averbach S, Curry T (University of California San Diego, United States); Kwan-Feinberg R, Consorti E, Gonzalez R, Grolman R, Liu T, Merzlikin O (Alta Bates Summit Medical Center (Sutter Health), United States); Abel MK, Ozgediz D, Boeck M, Kornblith LZ, Nunez-Garcia B, Robinson B, Park P (University of California San Francisco (UCSF), United States); Utria AF, Rice-Townsend SE, Javid P, Hauptman J, Kieran K (Seattle Children's Hospital, United States); Nehra D, Walters A, Cuschieri J, Davidson GH (Harborview Medical Center, United States); Nunez J, Cosker R (University of Utah Healthcare, Salt Lake City, United States); Eckhouse S (Washington University School of Medicine, United States); Choudhry A, Marx W (Suny Upstate University Hospital, United States); Jamil T, Seegert S, Al-Embideen S (ProMedica Toledo Hospital, United States); Quintana M, Jackson H (The George Washington University Hospital, United States); Wexner SD, Kent I (Cleveland Clinic Florida, Weston, United States); Martins PN (University of Massachusetts, UMass Memorial Hospital, United States); Alshehari M, Al-Naggar H, Alsayadi M, Alyazidi M, Shream S, Alhaddad W, Maqus A, Abu Hamra'a M, Alsayadi R, Ghannam R, Al-Maqtari S, Masdoos S, Al-Harazi Y, Bajjah H (Al-Thawra Modern General Hospital, Yemen); Al-ameri S, Aldawbali M (Royal Hospital, Yemen); Gwini GP (Mpilo Central Hospital, Bulawayo, Zimbabwe); Mazingi D (Parirenyatwa hospital, Harare, Zimbabwe).

## Funding

National Institute for Health Research (NIHR) Global Health Research Unit grant (NIHR 16.136.79) using UK aid from the UK government to support global health research, Association of Coloproctology of Great Britain and Ireland, Bowel & Cancer Research, Bowel Disease Research Foundation, Association of Upper Gastrointestinal Surgeons, British Association of Surgical Oncology, British Gynaecological Cancer Society, European Society of Coloproctology, NIHR Academy, Sarcoma UK, The Urology Foundation, Vascular Society for Great Britain and Ireland, and Yorkshire Cancer Research. The views expressed are those of the authors and not necessarily those of the National Health Service, NIHR, or UK Department of Health and Social Care.

*Disclosure.* The authors declare no conflict of interest.

## Supplementary material

Supplementary material is available at *BJS Open* online.

## Supplementary Material

zrab131_Supplementary_Data
